# Classical Force-Field Parameters for CsPbBr_3_ Perovskite Nanocrystals

**DOI:** 10.1021/acs.jpcc.2c00600

**Published:** 2022-06-01

**Authors:** Roberta Pascazio, Francesco Zaccaria, Bas van Beek, Ivan Infante

**Affiliations:** † Department of Nanochemistry, Istituto Italiano di Tecnologia, Via Morego 30, 16163 Genova, Italy; ‡ Dipartimento di Chimica e Chimica Industriale, Università degli Studi di Genova, Via Dodecaneso 31, 16146 Genova, Italy; § Department of Theoretical Chemistry, Faculty of Science, Vrije Universiteit Amsterdam, de Boelelaan 1083, 1081 HV Amsterdam, The Netherlands; ∥ BCMaterials, Basque Center for Materials, Applications, and Nanostructures, UPV/EHU Science Park, Leioa 48940, Spain; ⊥ Ikerbasque, Basque Foundation for Science, Bilbao 48009, Spain

## Abstract

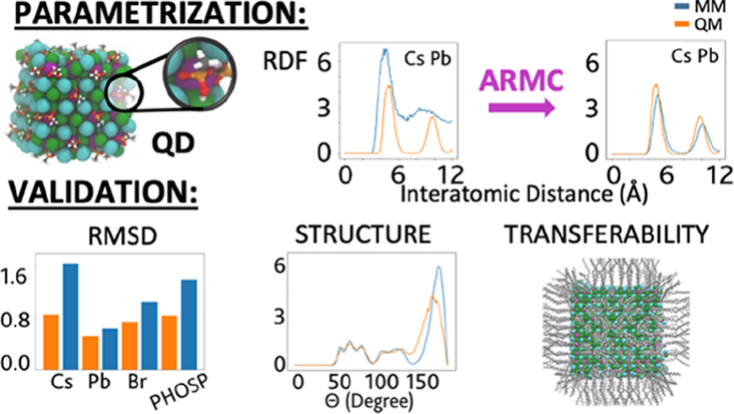

Understanding the chemico-physical properties of colloidal semiconductor nanocrystals (NCs) requires exploration of the dynamic processes occurring at the NC surfaces, in particular at the ligand–NC interface. Classical molecular dynamics (MD) simulations under realistic conditions are a powerful tool to acquire this knowledge because they have good accuracy and are computationally cheap, provided that a set of force-field (FF) parameters is available. In this work, we employed a stochastic algorithm, the adaptive rate Monte Carlo method, to optimize FF parameters of cesium lead halide perovskite (CsPbBr_3_) NCs passivated with typical organic molecules used in the synthesis of these materials: oleates, phosphonates, sulfonates, and primary and quaternary ammonium ligands. The optimized FF parameters have been obtained against MD reference trajectories computed at the density functional theory level on small NC model systems. We validated our parameters through a comparison of a wide range of nonfitted properties to experimentally available values. With the exception of the NC–phosphonate case, the transferability of the FF model has been successfully tested on realistically sized systems (>5 nm) comprising thousands of passivating organic ligands and solvent molecules, just as those used in experiments.

## Introduction

During the past two decades, research on colloidal semiconductor nanocrystals (NCs) has prompted new applications, ranging from photovoltaics and photocatalysis^1−12^ to optoelectronics,^13−23^ thanks to their solution processability and remarkable versatility. The primary requirement of these materials is the presence of a photoactive region, represented by a nanometer-sized inorganic semiconductive crystalline core, surrounded by organic ligands that maintain the NC colloidally stable. Examples of colloidal NCs include binary metal chalcogenides, pnictogenides, and ternary perovskites. In particular, lead-based perovskites with the chemical formula APbX_3_ (A being a large monovalent organic or inorganic cation, like methylammonium or Cs, respectively, and X being a halide anion) are regarded as highly efficient materials,^24^ containing promising electronic and photophysical properties such as high photoluminescence quantum yields (PLQYs).^25−28^ A comprehensive understanding of colloidal NCs should not neglect the atomistic processes occurring at their surfaces and how these influence their optoelectronic characteristics. Over the past few decades, computational research endeavors in this field helped in understanding, among many other things, how trap states emerge at NC surfaces and the importance of including explicitly ligands in the simulation. These computational works usually focus on the investigation of ground-state electronic and structural features of NC systems by employing realistic NC models that can be described at various levels of theory, most notably density functional theory (DFT).^29−33^ However, the high computational cost of DFT sets a definite upper boundary to the feasibility of NC models, both in terms of their dimensions (reaching at most 3–4 nm)^34^ and simulation time spans (usually no longer than 5 ps with molecular dynamics simulations).^35,36^ If one wants to include ligands and solvents, as it is done in experiments, and understand with greater detail surface processes, then a computationally much cheaper alternative to *ab initio* simulations must be chosen. A valid approach is indeed based on classical MD simulations,^35−37^ which utilize classical potential energy functions (force fields, FF). Historically speaking, FFs have been utilized extensively for (bio-)organic molecules,^38,39^ while only minor endeavors have attempted to use them for inorganic materials. This is largely a consequence of FF parameters that are usually insufficiently accurate for inorganic elements and thus have a limited availability.

In this work, we optimized FF parameters for CsPbBr_3_ perovskite NC models, including also the passivating ligands. In our procedure, the FF parameters are fitted against DFT references by means of a stochastic algorithm known as adaptive rate Monte Carlo (ARMC),^36^ which involves a comparison of nonfitted properties to their DFT-computed equivalents and a prediction of a range of structural properties available in the existing literature from both theoretical and experimental standpoints. We then demonstrated the scalability of the newly developed FF model on upscaled sized systems, mirroring those used in experiments.

## Computational Details

### NC Model

As a measure to ensure effective solubilization, NCs are synthesized by a nucleation and growth process in a setup involving the reaction of molecular precursors, *i.e.*, metallo-organic species where the organic part is composed of long-tail organic molecules, whose anchoring groups ultimately contribute to capping the surface of the NC inorganic core. High ligand coverages are known to maximize the stability of the NC, reducing the formation of trap states and increasing the photoluminescence quantum yields of these materials.^31,40−43^ In particular, CsPbBr_3_ NCs are usually capped with anionic and cationic ligands containing long aliphatic chains, such as oleate^44^ and oleylammonium molecules, as well as quaternary ammonium bromide (*e.g.*, DDAB).^25^ Sulfonic^45^ and phosphonic acid^41^ ligands, most notably oleylphosphonic acids (OLPA) and dodecylbenzene sulfonic acid (DBSA),^46^ have also been regarded as feasible candidates for the capping of perovskite cores due to their affinity for Pb^2+^ cations. Further attempts by Krieg *et al*.^45^ have also suggested that the capping of perovskite cores by zwitterionic ligands is an effective strategy in enhancing their PLQY and increasing their stability in apolar solvents.

A computational investigation of these materials, to reproduce what is done in experiments, should thus include both ligands, solvent molecules and realistically sized NC inorganic core models. In the case of classical FF approaches, paramount is the accuracy of those parameters that enter the potential energy function. Past research endeavors have successfully developed FFs targeted at the description of noncovalent interactions between inorganic ions in bulk hybrid organic/inorganic perovskite materials,^35,47−51^ where the charges entering the Coulombic term were fitted against *ab initio* computed data. However, the inherently high surface/volume ratio in NCs necessitates accounting for the surface, which is not included in bulk solids.^40,52^ This brings us to a total of four contributions necessary to parametrize NCs:

1. the interaction among the ions/species of the NC core,
2. the ligand–NC interactions, in particular of the ligand anchoring group directly attached to the NC surface,
3. the interaction of the surface with the ligand atoms located on the more distant, apolar portions of the organic tails, and
4. the interligand/ligand and ligand/solvent interactions.

**Scheme 1:**
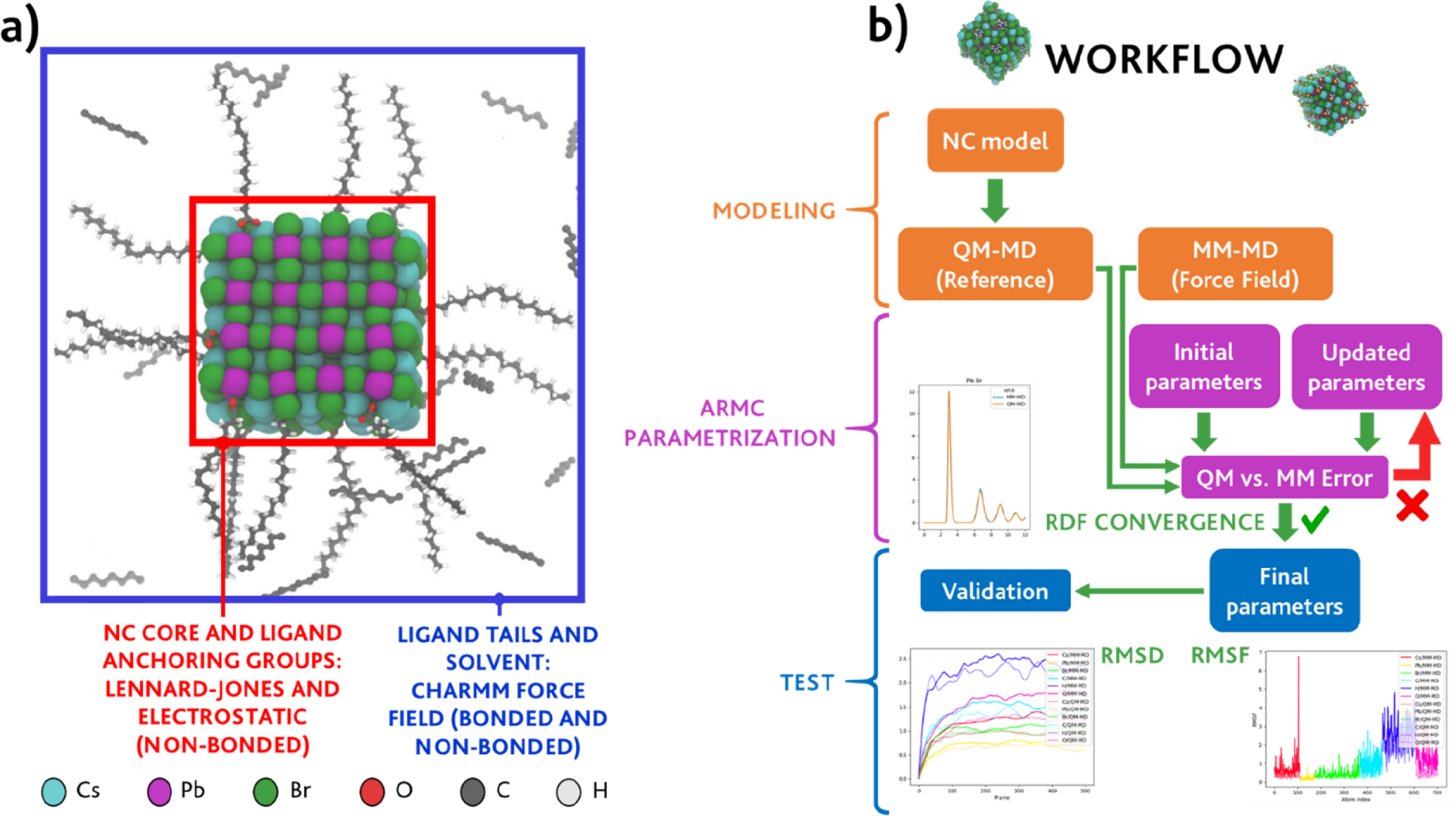
(a) Different Force-Field Components (Interactions) and Respective Descriptions in an Oleate-Capped CsPbBr_3_ NC Solvated in Octadecene and (b) Adaptive Rate Monte Carlo Parametrization Workflow

For the last point, an FF is required that describes known organic solvents and ligands, typical molecules for which bonded and nonbonded parameters are mostly available via FFs like CHARMM^53^ or AMBER.^54,55^ In the case of organic species for which FF parameters are not directly available, we will rely on the MATCH program,^56^ which automatically generates CHARMM-based atom types and FF parameters by comparison of molecular fragments. Furthermore, while the long-range energetic contributions of point 3 above are marginal, excluding them in their entirety possesses the risk of the NC and ligand tails to collapse due to the absence of a repulsive potential. For this reason, we decided to generate the Lennard-Jones parameters acting between the NC core and the organic tails using the combination rule on (comparatively) less accurate force-field parameters that span the entire periodic table (*e.g.*, a UFF^57^). Finally, over the course of this work, we focus our attention to points (1) and (2), *i.e.*, to parametrize the pair-specific interactions between the inorganic core ions and between the inorganic core and the anchoring group of the ligands bound to the NC surface. Once again, this choice can be motivated by the demand to simultaneously maximize the precision of the calculation and minimize the risk of the overfitting of the model. A schematic view of our strategy is depicted in Scheme 1a.

### Force-Field Model

The interactions between the NC core ions and between the NC core and the anchoring group of the ligands are represented by the effective pairwise-addictive potential:
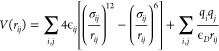
1The first term in the equation is the Lennard-Jones (LJ) 12-6 potential, encompassing both the van der Waals interactions and the Pauli repulsions: here, the well depth and the interatomic distance are respectively accounted for by the terms ε_*ij*_ and σ_*ij*_. The second term measures the Coulombic electrostatic interaction acting between the point charges *q* of the *i*th and *j*th atoms. This potential energy function has already delivered promising results in earlier parametrizations by Rabani^50^ and by Cosseddu and Infante^36^ on CdSe NC models, providing a good compromise between performance and accuracy. An important feature of this model is that the NC–ligand description neglects covalent contributions that would otherwise bind the ligands to their anchoring sites, decreasing the overall flexibility of the FF. Within the expression in (1), the dominant parameters are the charges and the σ_*ij*_ terms, whereas the ε_*ij*_ parameters are kept constant and precomputed at the universal force-field (UFF)^57^ level of theory, as it was observed that variations in ε_*ij*_ have little effect on the quality of the fit (*vide infra*). As an additional measure, the parameters employed in the FF model to treat the interior atoms and the surface atoms of the NC are identical. Although this may seem at odds with chemical intuition, this choice is motivated by the need to account for three features:

1. the transferability of the potential energy functions on larger-scaled systems,
2. the presence of ion exchange reactions between the core and surface atoms of the NC that usually occur in experiments, and
3. a reduction of the number of parameters to be fitted that prevents issues connected to overfitting.

### Force-Field Parametrization

The main assumption at the basis of our FF parametrization procedure is that all physical properties calculated by means of classical molecular mechanics (MM) are expected to converge to those calculated with a reference *ab initio* method. A crucial requirement is therefore the choice of the property onto which the parameters are fitted. To this aim, we decided to focus on the radial distribution function (RDF), *i.e.*, the probability to find a pair of atoms *i* and *j* at a given distance *r_ij_*. The RDF has the advantageous characteristic that it directly maps, via a Boltzmann inversion, to the potential of mean force *F* acting between a pair of atoms:^58^
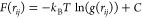
2where *g*(*r_ij_*) is the RDF, *k*_B_ is the Boltzmann constant, *T* is the temperature of the system, and *C* is a generic constant. The error function itself consists of two terms: a reference QM potential energy surface descriptor (*i.e.*, the RDF) and the to-be-fitted MM counterpart. In eq 3, this is represented by *N* vector pairs *g*^QM^ and *g*^MM^ (one RDF for each atom pair), each consisting of *M* grid points on which the RDF is evaluated:
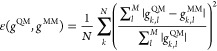
3We decided to fit the RDF and not *F* itself because fitting the latter would result in infinite values for those points of the potential energy surface only little sampled during the reference DFT trajectory. RDF plots, on the other hand, give out zero values for the same points, thus preventing divergence of the error function. The workflow for our FF parametrization is implemented through the following steps, which are represented schematically in Scheme 1b: a reference NC model is prepared, and an MD trajectory 5–10 ps-long is calculated using density functional theory (DFT; see the Computational Details section).^59^ An initial set of FF parameters is guessed to run a preliminary MM-MD simulation, and the error function is computed. The classical FF parameters are then updated on the fly through a specific procedure, known as the adaptive rate Monte Carlo^36^ (ARMC) algorithm, an automated stochastic procedure. The calculation is manually stopped once a sufficiently accurate set of parameters has been acquired.

The overfitting of the model is averted with the help of an extra validation set consisting of angular distribution functions (ADFs), root-mean-square fluctuation and displacement (RMSF and RMSD), bulk modulus, and Young’s modulus. These properties are compared to both *ab initio* computed and experimentally available values.^60−63^ In addition, the risk of overfitting is reduced by limiting the size of the parameter space. This is accomplished via the inclusion of charge constraints (*vide infra*), as well as a reduction in the number of fitting parameters (*i.e.*, some parameters are set as constant in the model) while retaining an overall low error value.

### Preparation of the NC Models

**Figure 1:**
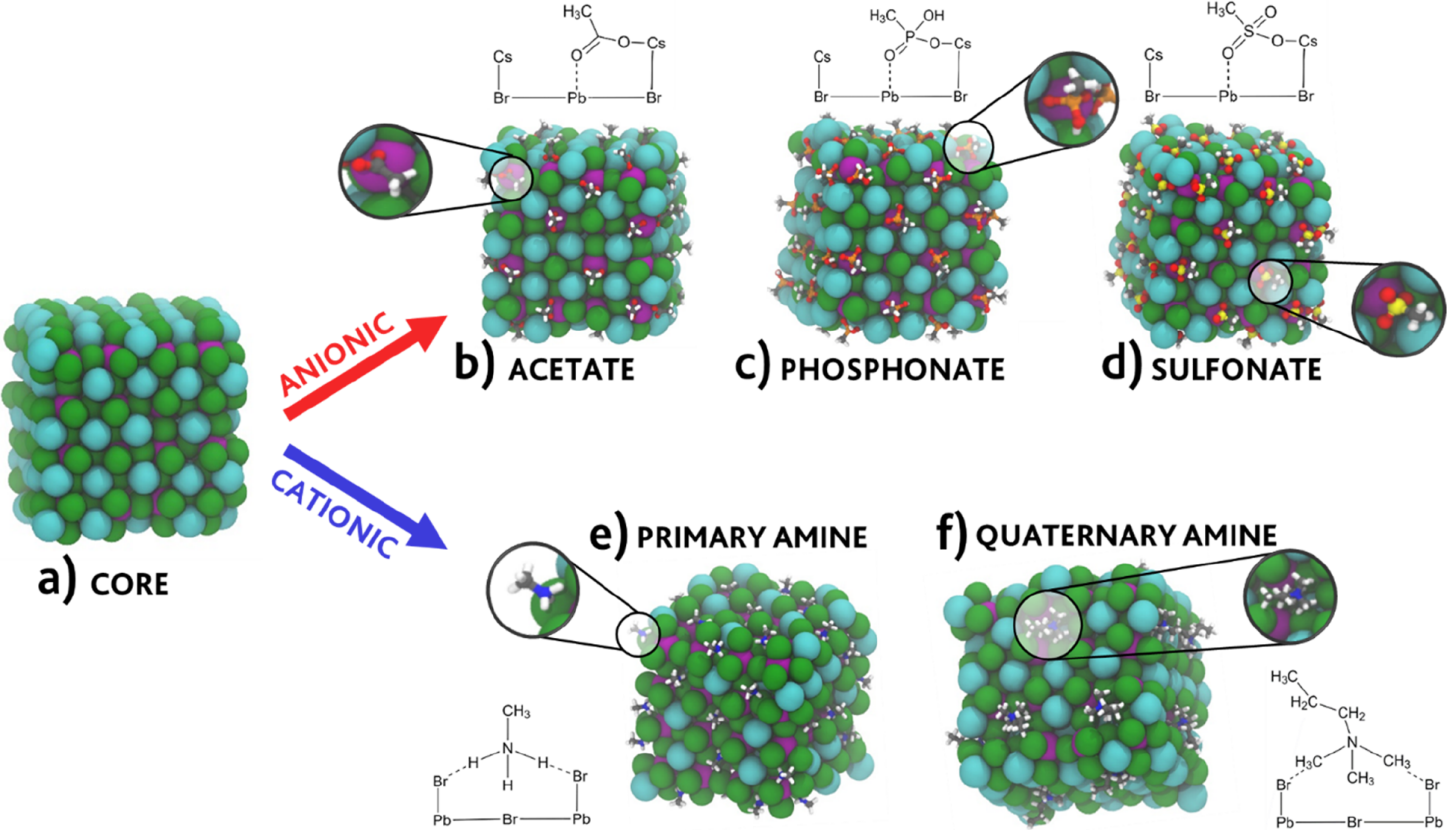
Snapshots of the NC models parametrized in this work: (a) inorganic CsPbBr_3_ core and nanocrystal models capped with (b) acetate, (c) methylphosphonate, (d) methylsulfonate, (e) methylammonium (a primary amine), and (f) trimethyl propylammonium (a quaternary amine).

A NC model presenting a 2.3 nm diameter was built by cutting the facets of a cubic CsPbBr_3_ lattice (space group *Pm*3̅*m*) through the (001) directions so that the layer containing Cs and Br atoms would be exposed to the surface. This choice shows good agreement with experimentally available information on the Pb:Br molar ratios that strongly suggest a CsBr type of termination,^16,17^ and it has already been successfully applied in previous computational simulations involving metal halide perovskite cores.^31,33,64^ Cs cations were additionally removed from the surface of the inorganic core in order to achieve a neutral, charge-balanced Cs_112_Pb_64_Br_240_ model. Organic ligand molecules in an ionic form were then set to replace a given percentage, *i.e.*, coverage, ranging from 14 to 38%, of surface ions of the same overall charge (*i.e.*, Br for anionic ligands and Cs for cationic ligands), as to preserve the neutrality of the model (see later for further details on the NC–ligand systems). Overall, a total of six NC–ligand models were fitted in this work: one CsPbBr_3_ inorganic core without ligands; three CsPbBr_3_ models passivated with anionic ligands, *i.e.*, acetate, methylphosphonate, and methylsulfonate; and CsPbBr_3_ models passivated with the cationic ligands methylammonium (a primary amine) and trimethyl propylammonium (a quaternary amine). Short organic moieties (*i.e.*, methyl and isopropyl groups) were purposely used for the NC models as to minimize the computational load of the reference DFT calculations. The selected anchoring groups are representative of the most commonly employed ligands in experiments.^41,65−70^ A representation of the investigated systems is provided in Figure 1.

### Simulation Details

*Ab initio* QM-MD simulations have been conducted on the aforementioned NC models at the DFT/PBE/DZVP level of theory, utilizing the Perdew–Burke–Ernzerhof (PBE) exchange–correlation functional^71^ and a double-ζ basis set with polarization functions (DZVP) as implemented in the CP2K 6.1 quantum chemical package.^72,73^ This level of theory is regarded as an adequate choice for the description of the properties and features of interest in other families of semiconductor NCs.^74^ The ARMC parametrization procedure was then executed using the Auto-FOX package^75^ to fit the FF parameters. For each set of parameters, MM-MD simulations were run with a smooth particle mesh Euler method (SPME) using beta-Euler splines^76^ for the computation of the LJ and Coulombic terms.

The spacing between consecutive grid points was set to be 0.62 Å. For both the QM-MD simulations and the classical MM ones, cubic cells of 40 and 100 Å, respectively, have been chosen, purposely exceeding the NC dimensions and thus leaving vacuum on the sides of the simulation box. The integration steps were chosen to be 2.5 fs when involving the NC core without ligands, while a 1 fs time step was chosen for the ligand-capped NCs. The simulations were run while keeping a constant particle number *N*, constant volume *V*, and constant temperature *T* (NVT, canonical ensemble). The temperature of 300 K was maintained constant in the simulations by means of a velocity-rescaling thermostat.^77^ Canonical simulations were run for 10,000 steps, resulting in MD simulations ranging from 10 to 25 ps in total.

### Force-Field Parameters

The sets of charges and LJ parameters in eq 1 that have been fitted over the course of this work satisfy a set of constraints that are described below.

#### The Point Charges (*q*) of the NC Core

For our perovskite model, CsPbBr_3_, the charges have been forced to preserve an overall Cs:Pb:Br ratio of 1:2:–1 according to their most stable oxidation states, *i.e.*, +1 for Cs, +2 for Pb, and −1 for Br in our CsPbBr_3_ model. These constraints have been applied to the parameters, ensuring that in the case of the detachment of ionic couples from the surface of the core (*e.g.*, CsBr), both the NC and the detached moiety would preserve an overall neutral charge, improving the transferability of the parameters to larger sizes.

#### The Charges (*q*) of the Ligands

The total charge of each ligand was constrained to balance that of the atom that it was replacing (Br for anionic monovalent ligands and Cs for their cationic counterparts). As an additional measure, only the charges in the anchoring groups, closer to the inorganic core and thus accounting for stronger electrostatic interactions, were fitted. The remaining charges, *i.e.*, those of the atoms located on the ligand tails, were kept frozen (*i.e.*, their values were not optimized). Their fixed values were taken from the well-established CHARMM FF^53^ using the MATCH program.

#### The LJ Parameters (ε and σ) of the NC

The well depth (ε) and interatomic distances (σ) of the atom pairs of interest providing a description of both the interactions in the inorganic core (*e.g.*, Cs–Cs and Pb–Br) and those between the NC core and the organic anchoring groups of the ligands (such as, for instance, Br–C and Cs–O) were fitted, whereas the remaining terms, *i.e.*, those between the core atoms and the distant ligand tails account for marginal energetic contributions and were kept fixed. In addition, because the potential is dominated by electrostatic and short-repulsive interactions (*i.e.*, the sigma term), we considered the well depth epsilon value of secondary importance. Indeed, even a large change around a precomputed value at the UFF level of theory has little effect on the shape of the RDF. The initial values for these parameters were guessed from the DFT-computed RDFs of the individual atom pairs. Additionally, milder constraints were applied to the interatomic distances as a measure to ensure that their values did not fall within the repulsive region of the Lennard-Jones energy well, disrupting the stability of the systems.

## Results and Discussion

### Force-Field Fitting

**Figure 2:**
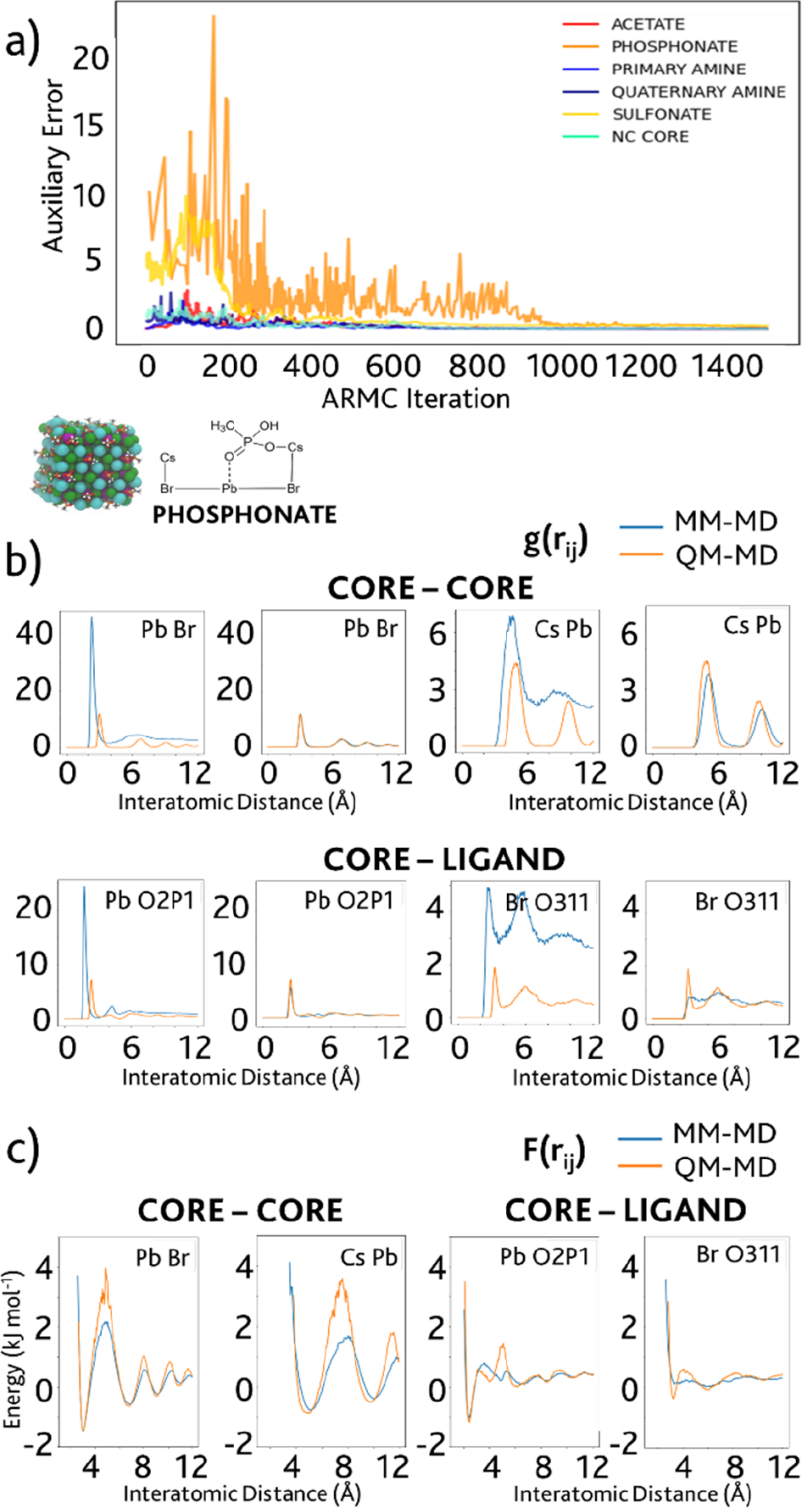
(a) Relative errors obtained for the accepted MD iterations during the ARMC fitting, over a maximum of 1500 iterations. (b) Example of the comparison between *ab initio* computed RDFs (QM-MD, orange line) and classical RDFs (MM-MD, blue line), computed using the ARMC-optimized FF parameters, for the phosphonate-capped NC model at the first iteration and at the best iteration. (c) Comparison between the QM (orange line) and MM (blue line) potential of mean force computed using eq 2 from the provided RDFs. Altogether, the following ligand charges have been taken into account (in parentheses are the name types according to CHARMM FFs): for carboxylate-based acetate, the carboxylic C (C2O3) and the two oxygens (O2D2); for phosphonate, P (PG1), the two O atoms connected by a single bond (O2P1) and the oxygen connected by a double bond (O311); the charges of the sulfur atom (S3O1) and of the negatively charged oxygen atoms bound to it (O2S1) for sulfonate; the nitrogen atom (N3P3) and the hydrogens (HGP2) in the primary amine and nitrogen (N3P0) and hydrogen (HGP5) charges, as well as those of the adjacent carbon atoms C334 for the methyl and C324 for the methylene bound to the propyl group for the quaternary ammonium cation, trimethyl propylammonium.

As a first step, we decided to optimize the FF parameters characterizing only the inorganic core, formally taking a NC without ligands. The RDFs obtained using the MM-computed FF parameters displayed an excellent convergence toward the reference DFT plots, with errors as low as 0.035 for the best set of parameters (Figure 2a). This preliminary optimization served as a tool to obtain starting parameters for the successive, more complex parametrizations of the NC–ligand systems. In the latter case, the best results, *i.e.*, those associated with the lowest relative errors, obtained for the potential energy function are presented in Table 1.

**Table 1:** Calculated Force-Field Parameters for the CsPbBr_3_ Perovskite NC Core and the Ligand-Capped NC^a^

	NC core	acetate		phosphonate		sulfonate		primary amine		quaternary amine	
Charges (NC core)											
Cs	0.5068	0.5519		0.7794		0.5180		0.6354		0.4678	
Pb	1.0136	1.1038		1.5588		1.0360		1.2708		0.9356	
Br	–0.5068	–0.5519		–0.7794		–0.5180		–0.56354		–0.4678	
σ (NC core)											
Cs Cs	0.462	0.541		0.370		0.489		0.442		0.438	
Cs Pb	0.354	0.390		0.364		0.329		0.3657		0.372	
Cs Br	0.396	0.372		0.398		0.394		0.396		0.378	
Pb Br	0.306	0.303		0.327		0.314		0.314		0.309	
Pb Pb	0.604	0.643		0.632		0.621		0.632		0.620	
Br Br	0.420	0.386		0.389		0.379		0.405		0.373	
Charges (ligand anchors)											
		O2D2	–0.4143	O2P1	–0.7381	O2S1	–0.2675	N3P3	–0.2792	N3P0	–0.2806
		C2O3	0.3766	O311	–0.6179	S3O1	0.3845	HGP2	0.2482	C334	–0.1637
				PG1	1.4247					C324	–0.047
				HGP1	0.3600					HGP5	0.1170
σ (ligand anchors to the NC core)											
		Cs C2O3	0.425	Cs O2P1	0.353	Cs O2S1	0.249	Cs N3P3	0.498	Cs N3P0	0.465
		Pb C2O3	0.291	Pb O2P1	0.281	Pb O2S1	0.270	Pb N3P3	0.384	Pb N3P0	0.560
		Br C2O3	0.288	Br O2P1	0.316	Br O2S1	0.395	Br N3P3	0.353	Br N3P0	0.448
		Cs O2D2	0.331	Cs O311	0.341	Cs S3O1	0.470	Cs HGP2	0.349	Cs C334	0.327
		Pb O2D2	0.275	Pb O311	0.212	Pb S3O1	0.296	Pb HGP2	0.288	Pb C334	0.384
		Br O2D2	0.361	Br O311	0.343	Br S3O1	0.347	Br HGP2	0.144	Br C334	0.360
				Cs PG1	0.411					Cs C324	0.380
				Pb PG1	0.228					Pb C324	0.380
				Br PG1	0.393					Br C324	0.271
				Cs HGP1	0.136					Cs HGP5	0.277
				Pb HGP1	0.335					Pb HGP5	0.265
				Br HGP1	0.223					Br HGP5	0.196

a Charges (*q*) are provided in elementary charge units, while the distances (σ) are reported in nm. Units are the default ones employed by the CP2K package.

Once again, the RDFs obtained with the MM-fitted values display very good agreement with the DFT reference: the best set of parameters is obtained for the NC model capped by the primary amine where the computed errors reached a minimum of 0.015. A representation of the trend of the error function for the core parameters and those of the NC models is presented in Figure 2a. Figure 2b, on the other hand, provides a comparison between the RDF plots achieved with the best parameters and the reference ones computed by DFT. Additionally, eq 2 was applied to the RDFs to compute the potential of mean force of the different core atom pairs. Examples of the plots obtained of these functions, once again displaying a agreement with the DFT reference plots, are shown in Figure 2c.

A less accurate set of parameters, on the other hand, was found for the NC model containing phosphonate ligands. We attribute the inaccuracies found in this NC model to two reasons: (1) a low quality of the MATCH FFs for the bare phosphonate ligand as evidenced by a discrepancy on the relaxed structure of the ligand alone obtained with DFT and the default MATCH parameters; (2) the oversimplified FF based on the Lennard-Jones and Coulombic terms only, whereas the polarization contribution may further improve the interaction between the anchor and the NC surface ions. This aspect will be analyzed with greater detail in the future.

In addition to the above, our fitting scheme allows for a further refinement of the FF parameters, for example, by fitting simultaneously multiple DFT-MD trajectories where the NC is capped by different types of ligands. We provided an example of such simultaneous optimization by fitting the acetate-capped and the methyl ammonium-capped DFT trajectories at 300 K in the Supporting Information (see Table S9) considering that in the experiments, a CsPbBr_3_ NC is passivated with both oleate and oleylammonium. In this fit, the charge neutrality of the system is ensured by the previously established constraints: the ratios between the atom charges in the inorganic core are preserved, and the charges of the ligands are set to balance the atoms that they are replacing (Br for acetates and Cs for primary amines).

### Validation of the Method

**Figure 3:**
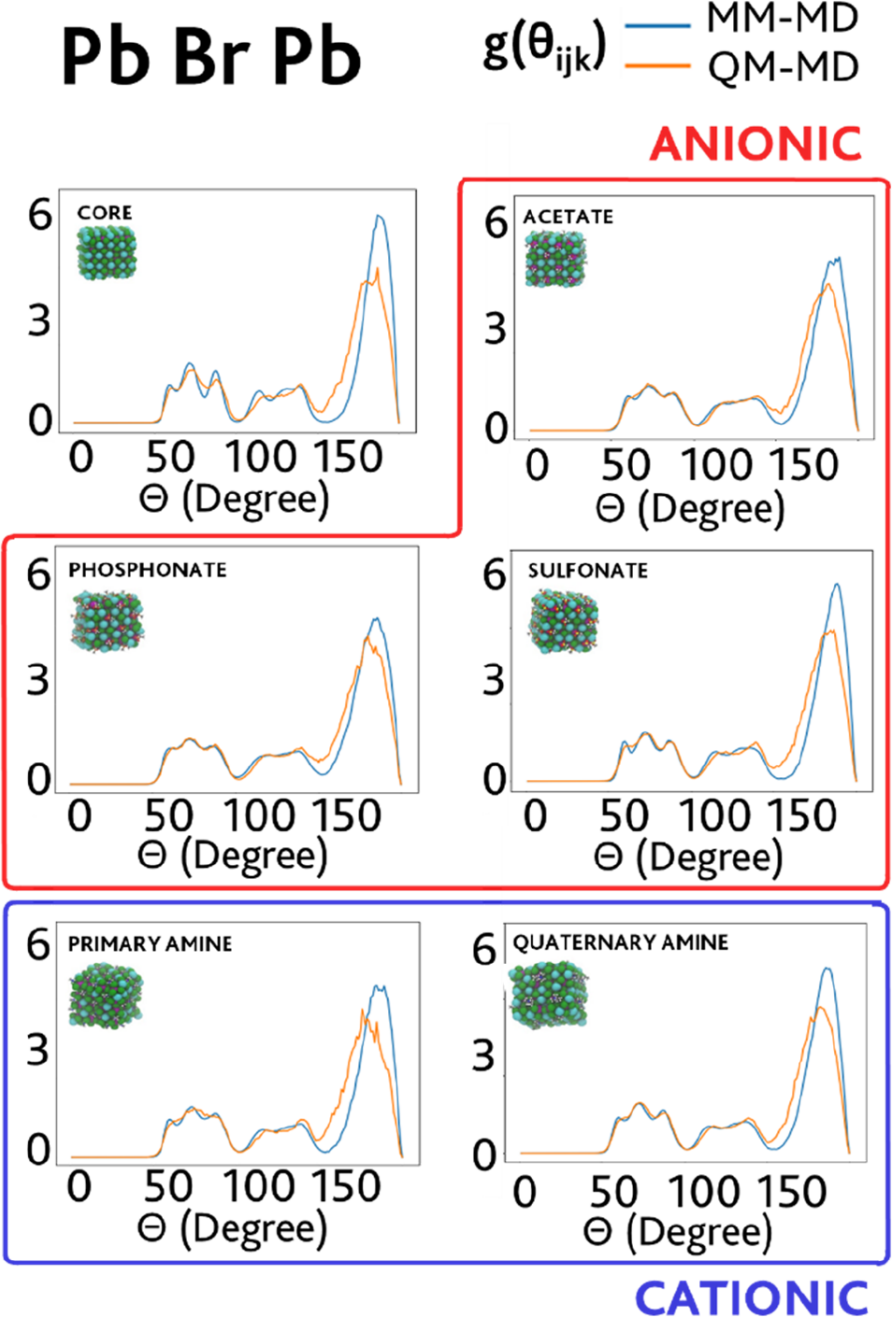
Comparison between *ab initio* computed ADFs (QM-MD, orange line) and classical ADFs (MM-MD, blue line) for the Pb–Br–Pb angle (between two nearby octahedra), predicted using the best ARMC-optimized FF parameters, for the inorganic core and the ligand-capped NC models.

The validation of the newly optimized potential energy function, *i.e.*, the proof of concept of the accuracy of the fitting, can be divided into two main approaches: (1) the validation of MM-calculated nonfitted properties to their DFT-computed equivalents; (2) the prediction of structural properties of the material in comparison to available experimental values. In the first step, we predicted the MM angular distribution functions *g*(θ_*ijk*_) (ADFs), *i.e.*, the measure of the probability to find three atoms *i*, *j*, and *k* at a given angle θ_*ijk*_, for the angle formed between two lead atoms and one bromine atom in the model structures, *i.e.*, between adjacent octahedra in the crystal lattice. In agreement with experimental findings,^78,79^ previous computational investigations on CsPbBr_3_ models had highlighted a deviation from the 90° angle corresponding to the cubic structure toward an orthorhombically distorted crystal structure.^31,80^ This trend, correctly represented in the reference DFT angular distribution functions, is indeed well-reproduced by the plotted MM functions in Figure 3.

**Figure 4:**
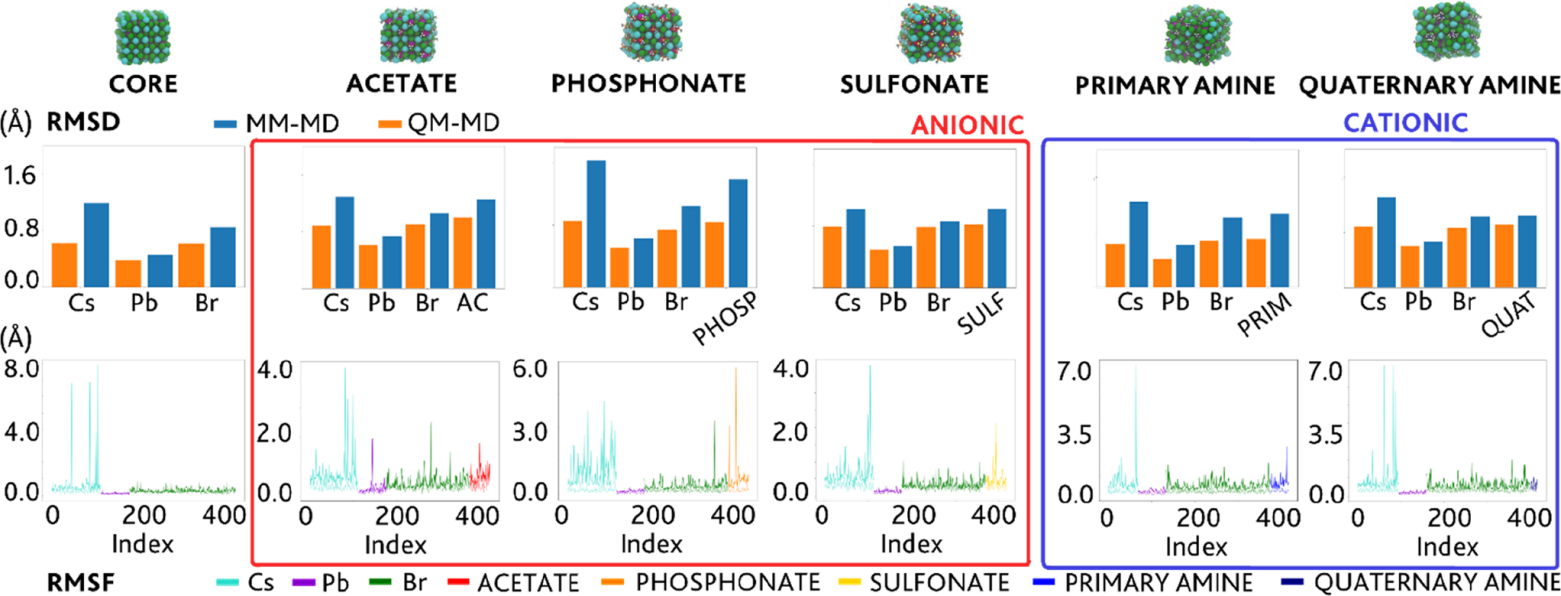
RMSD and RMSF plots: comparison between *ab initio* computed (QM-MD) and classically predicted properties using the best set of parameters fitted with the ARMC procedure (MM-MD), for the inorganic core and the ligand-capped NC models.

As an additional validation, we calculated the root-mean-square displacement (RMSD) for each atom type and its time-averaged equivalent, known as the root-mean-square fluctuation (RMSF). These functions are a measure of the spatial extent of the motions of the atoms, and the comparison between these classically derived functions and their DFT equivalents is expected to provide similar average values if the dynamical properties of the system are accurately represented by the FF. The DFT- and MM-based RMSD and RMSF plots are provided in Figure 4, and they indeed show good agreement, further outlining the effectiveness of the FF-based representation in the MD simulations. A similar procedure has been employed for the calculations of the binding energies of the ligand–ion couples to the entire NC model. The calculated values, provided in the Supporting Information of this manuscript (Table S8), show only qualitative agreement between the DFT- and MM-calculated energies. The discrepancies between the DFT and the MM energies range from a maximum discrepancy for phosphonate (∼26 kcal/mol) to the 0.7 kcal/mol deviation found for the methylammonium case. We must point out, however, that a good description of the binding energies needs to correctly account for the individual ligand–ion couples; thus, a balanced set of FF parameters can be obtained by including in the fitting protocol both the NC–ligand species (reactants) and ligand–ion couples (products).

To further improve our validation, Auto-FOX has been employed in conjunction with another package, TRAVIS,^83−86^ for the calculation of the power spectra of the systems obtained from the Fourier transform of the autocorrelation functions of the atomic velocities (VACF), *i.e.*, their vibrational densities of states (vDOS). These quantities can in fact be obtained from the DFT-MD trajectories and the MM-MD trajectories. The resulting spectra projected on the core atoms and the ligands are provided in the Supporting Information. The comparison between the DFT results and their classical equivalents has resulted in good agreement between the main peaks of the vDOS. The biggest uncertainties were found for the spectra of the atomic ligands; however, these can be attributed to the force constants of the atoms in the ligand tails that are not optimized here and have been retrieved from the literature (CHARMM force fields). The follow-up step in the validation of the ARMC fitting of the NC models involves the evaluation of several mechanical properties of the material. The Voigt–Reuss–Hill average of the bulk and Young’s moduli was computed by applying the newly optimized FF parameters for a bulk CsPbBr_3_ lattice using the GULP software.^81^ Given the intrinsically anisotropic nature of the Young’s modulus, the values in Table 2 account for predictions and/or measurements occurring on the (101) face of the bulk structure. As an additional comparison, the bulk modulus was computed numerically and extrapolated by a nonlinear fitting of the Birch–Murnaghan equation of state (BMEOS).^87^
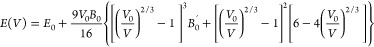
4

**Table 2:** Calculated Theoretical and Structural Properties for the CsPbBr_3_ Perovskite Bulk, the NC Core, and the Ligand-Capped NC: The Binding Energy (kcal mol^–1^), Bulk Modulus (GPa), and Young’s Modulus (GPa)^a^

Bulk Modulus (GPa)										
analytical value (GULP^81^ and VRH average^82^)								ref^60^	ref^61^	ref^62^
acetate	phosphonate	sulfonate	primary amine	quaternary amine	NC core	bulk		bulk	bulk	bulk
12.1	20.0	10.7	15.1	10.3	10.8	9.7		15.5	23.5	21

numerical extrapolation (BMEOS)^63^										
acetate	phosphonate	sulfonate	primary amine	quaternary amine	NC core	bulk	bulk and QM ref.			
12.9	22.4	12.1	10.5	15.1	10.9	11.2	14.2			

Young’s Modulus (GPa)										
analytical value (GULP^81^), the (101) face								ref^60^		
acetate	phosphonate	sulfonate	primary amine	quaternary amine	NC core	bulk		bulk		
11.4	20.9	8.8	11.5	7.1	16.7	14.3		15.8		

a Their units of measurement are the default ones employed by the GULP package:^81^ both properties are reported in GPa.

This equation is a function of the lattice energies *E* at different cell volumes *V*, *E*(*V*), for infinitesimal cell deformations, under the validity of Hooke’s law. *E*_0_ and *B*_0_ respectively account for the energy and the bulk modulus computed for the system at the reference volume *V*_0_, *B*_0_^′^ is the derivative of *B*_0_ with respect to the pressure. Once again, Table 2 provides a comparison of the computed values to the bulk modulus calculated from the DFT-optimized geometry of the bulk at different compression factors and to the experimental,^60^ computational,^61^ and semiempirical^62^ measurements available in the literature, outlining the excellent agreement between these data and values obtained using the different FF parameters. The deviations visible in the NC models depicting the ligand-capped models and the inorganic core, on the other hand, can be ascribed to the interplay of the ligands with the NC core in the finite-sized NC systems.^40,52^ This contribution modifies the inorganic core parameters ultimately affecting bulk and Young’s moduli.

### Transferability to Larger Sizes

**Figure 5:**
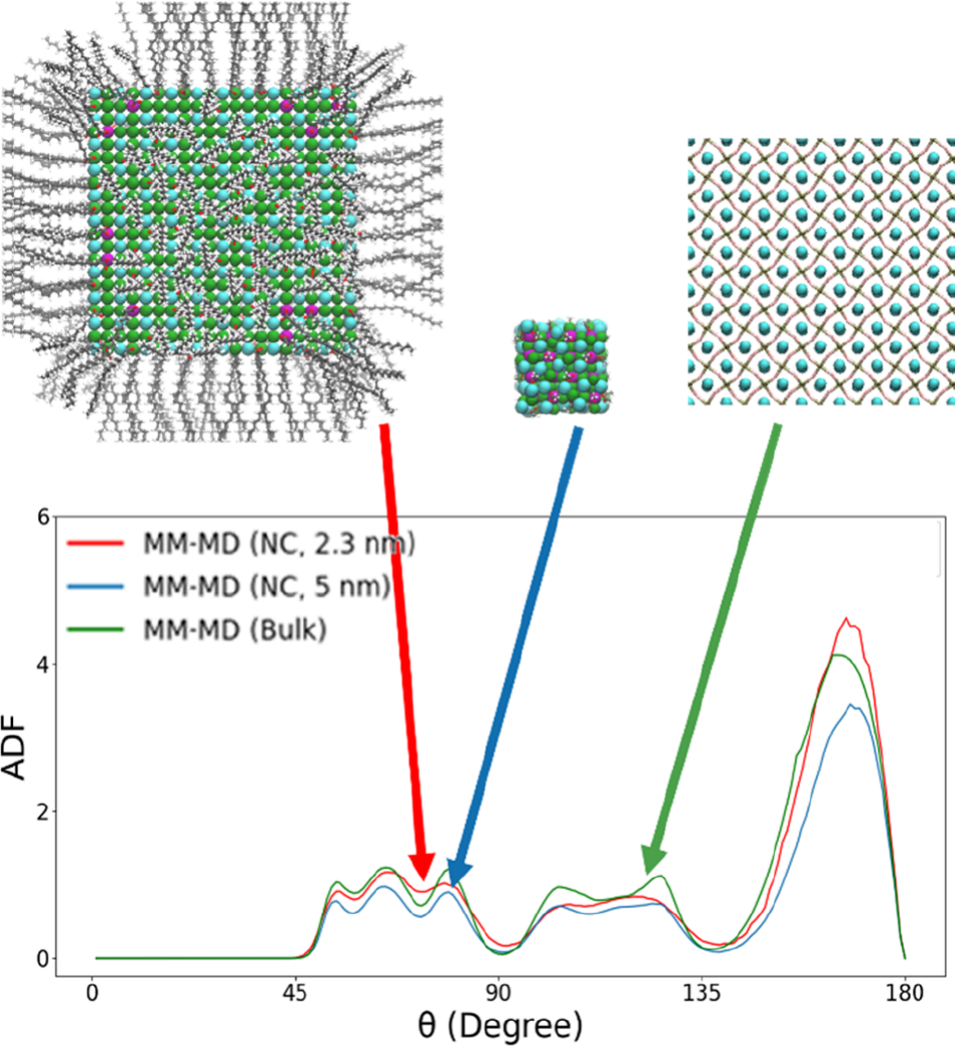
Comparison between the ADFs of the Pb–Br–Pb angle obtained for the downscaled 2.3 nm NC model capped by oleate ligands (blue plot), for the upscaled 5.0 nm NC model capped by oleate ligands (red plot) in vacuum, and for the bulk structure (green plot), plotted from MM-MD simulations obtained using the best ARMC-optimized FF parameters.

The last validation of our parametrization scheme has involved testing the scalability of the newly developed FF parameters on a more realistic model:^44^ a 5.0 nm-sized CsPbBr_3_ NC, CsBr-terminated, capped with typical long chain ligands employed in previously established synthetic protocols, namely, oleate (OA^–^),^25^ oleylammonium (OLAH^+^),^45^ didodecyldimethylammonium bromide (DDAB),^68,70^ oleylphosphonic acids (OLPA),^88^ and dodecylbenzene sulfonic acid (DBAS^46^), in longer MD simulations. In a parallel fashion to the downsized systems, anionic ligands were set to replace Br atoms and cationic ligands replaced Cs atoms to preserve the charge neutrality of the NC models. Other than the DDAB-capped NC, exhibiting a 40% coverage, which was deemed to be closer to the experimental findings for these systems,^31,89^ a 60% ligand coverage was chosen for all the remaining ligand-capped NC models. The simulations were run keeping an NVT ensemble, with a constant temperature of 300 K. Canonical simulations were run for one million steps of 1 fs each resulting in MD simulations of 1 ns in total. A comparison of the ADFs between the acetate-capped 2.3 nm CsPbBr_3_ core employed in the fitting procedure at the DFT level of theory, an upscaled equivalent oleate-capped 5.0 nm NC, and a CsPbBr_3_ bulk structure computed using the optimized FF parameters at the MM level of theory is provided in Figure 5. ADFs provide a measure of the preservation of the integrity of the orthorhombic nature both in the bulk structures and in the NC models, regardless of the dimension of the lattice. This feature, which has been proven experimentally by Cottingham and Brutchey^90^ and by ten Brinck and Infante^33^ through theoretical models, further corroborates the effectiveness of the newly developed FF in the prediction of the structural properties of CsPbBr_3_ NC models. Plots containing their average total energies (*i.e.*, the sum of their kinetic and potential energies) are provided in the Supporting Information, highlighting the achievement of a substantial equilibration of the energies and temperatures in the MD simulations and thus deemed to provide stable systems.

## Conclusions

Over the course of this work, we carried out FF parametrizations of the most common ligand-capped CsPbBr_3_ NC models, as well as of their bare inorganic core, based upon the stochastic adaptive rate Monte Carlo parametrization algorithm.^36^ We have consequently validated the robustness of our ARMC-optimized FF parameters via comparison with both computational (*ab initio*) as well as experimental data. The classical force fields obtained with the newly developed parameters provide an efficient, computationally feasible methodology for the description of the structural and time-dependent dynamics of perovskite NCs, paving the way toward long timescale simulations of more realistically sized systems, involving the explicit inclusion of solvent molecules as well as ligand chains. We tested several NC–ligand combinations, and we obtained good results for all with the exception of the NC–phosphonate case. Here, we speculate that the starting FF parameters of the phosphonate alone are already flawed; however, this aspect requires further investigations.

## Supporting Information Available

Comparison between the QM reference and MM-fitted RDFs and free energy plots, CHARMM frozen tail parameters (*q* and σ) and the trend of the to-be-fitted parameters (*q* and σ) during the FF optimizations, comparison between the QM reference and MM-fitted vDOS, average total (*i.e.*, sum of kinetic and potential) energies of the MM-MD simulations obtained for the upscaled NC models, example of yaml input and fitted parameters used for other fitting procedures (PDF)

## Abbreviations

DFT: density functional theory
PLQY: photoluminescence quantum yield
UFF: universal force field
FF: force field
MM: molecular mechanics
QM: quantum mechanics
ARMC: adaptive rate Monte Carlo
RDFs: radial distribution functions
ADFs: angular distribution functions
NCs: nanocrystals
LJ: Lennard-Jones
BMEOS: Birch–Murnaghan equation of state
