# Supplementary material for: Classical Force-Field Parameters for CsPbBr3 Perovskite Nanocrystals
Source: J Phys Chem C Nanomater Interfaces. 2022 Jun 1;126(23):9898–908. doi: 10.1021/acs.jpcc.2c00600 (PMC9207923; doi:10.1021/acs.jpcc.2c00600)
Supplement: Supplementary file 1 — jp2c00600_si_001.pdf [file jp2c00600_si_001.pdf]

Classical Force-Field Parameters for $CsPbBr_3$ Perovskite Nanocrystals

Roberta Pascazio[a,b], Francesco Zaccaria[a], Bas van Beek[c], Ivan Infante[a,d,e*]

[a]Department of Nanochemistry, Istituto Italiano di Tecnologia, Via Morego 30, 16163 Genova, Italy

[b]Dipartimento di Chimica e Chimica Industriale, Università degli Studi di Genova, Via Dodecaneso 31, 16146 Genova, Italy

[c]Department of Theoretical Chemistry, Faculty of Science, Vrije Universiteit Amsterdam, de Boelelaan 1083, 1081 HV Amsterdam, The Netherlands

[d]BCMaterials, Basque Center for Materials, Applications, and Nanostructures, UPV/EHU Science Park, Leioa 48940, Spain

[e]Ikerbasque Basque Foundation for Science Bilbao 48009, Spain

The plots and statistical analysis provided in the following sections were developed using the python based module Matplotlib[1], while the pictures were obtained using VMD[2].

S1. Radial distribution functions

The comparison between the classically fitted radial distribution functions (RDFs) and the quantum-mechanically computed reference RDFs are hereby represented for the models involved in the simulations is hereby provided in Figure S1.

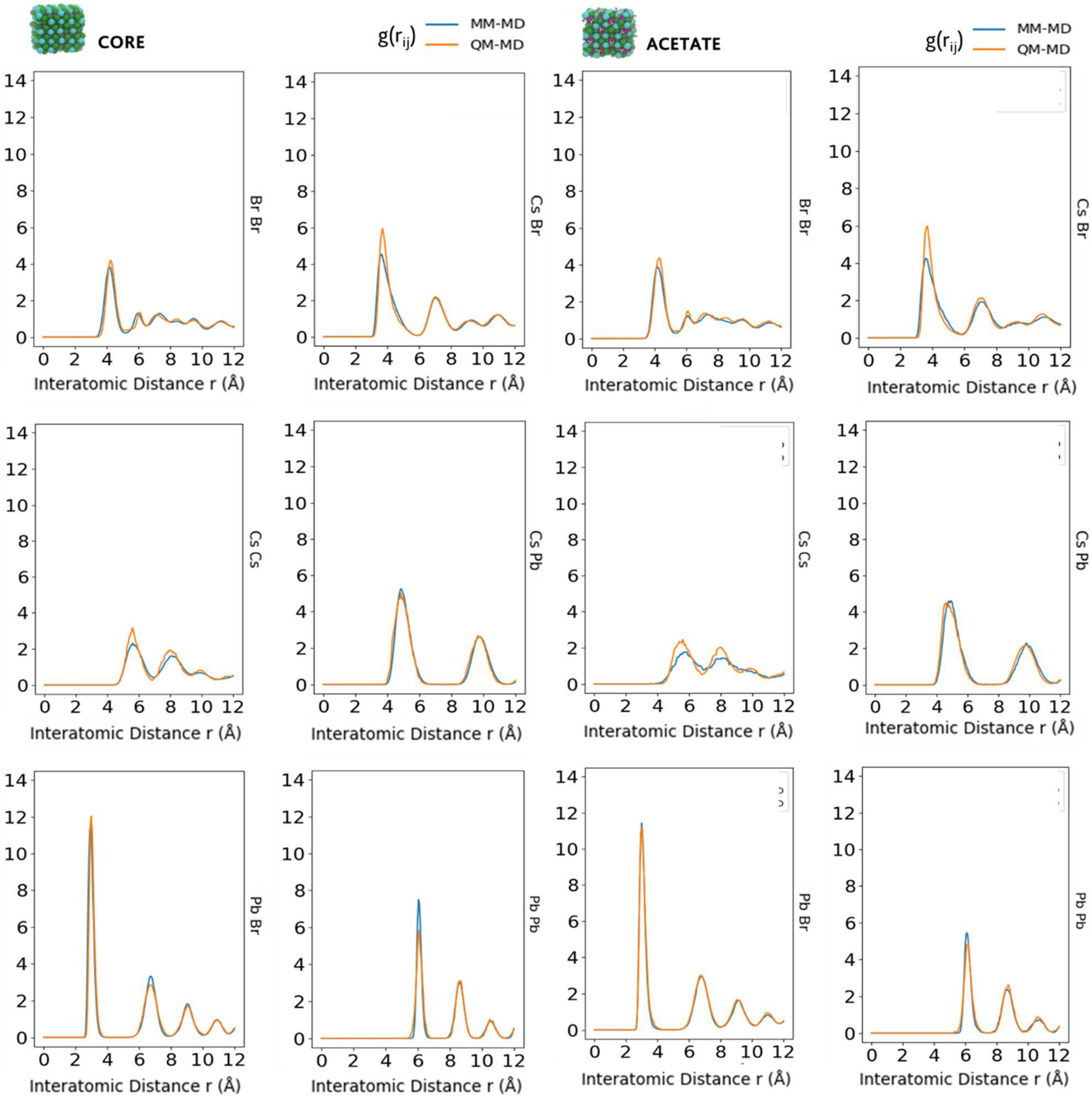
CORE
ACETATE
g(r_ij)
MM-MD
QM-MD
Br Br
Cs Br
Cs Cs
Cs Pb
Pb Br
Pb Pb
Interatomic Distance r (Å)

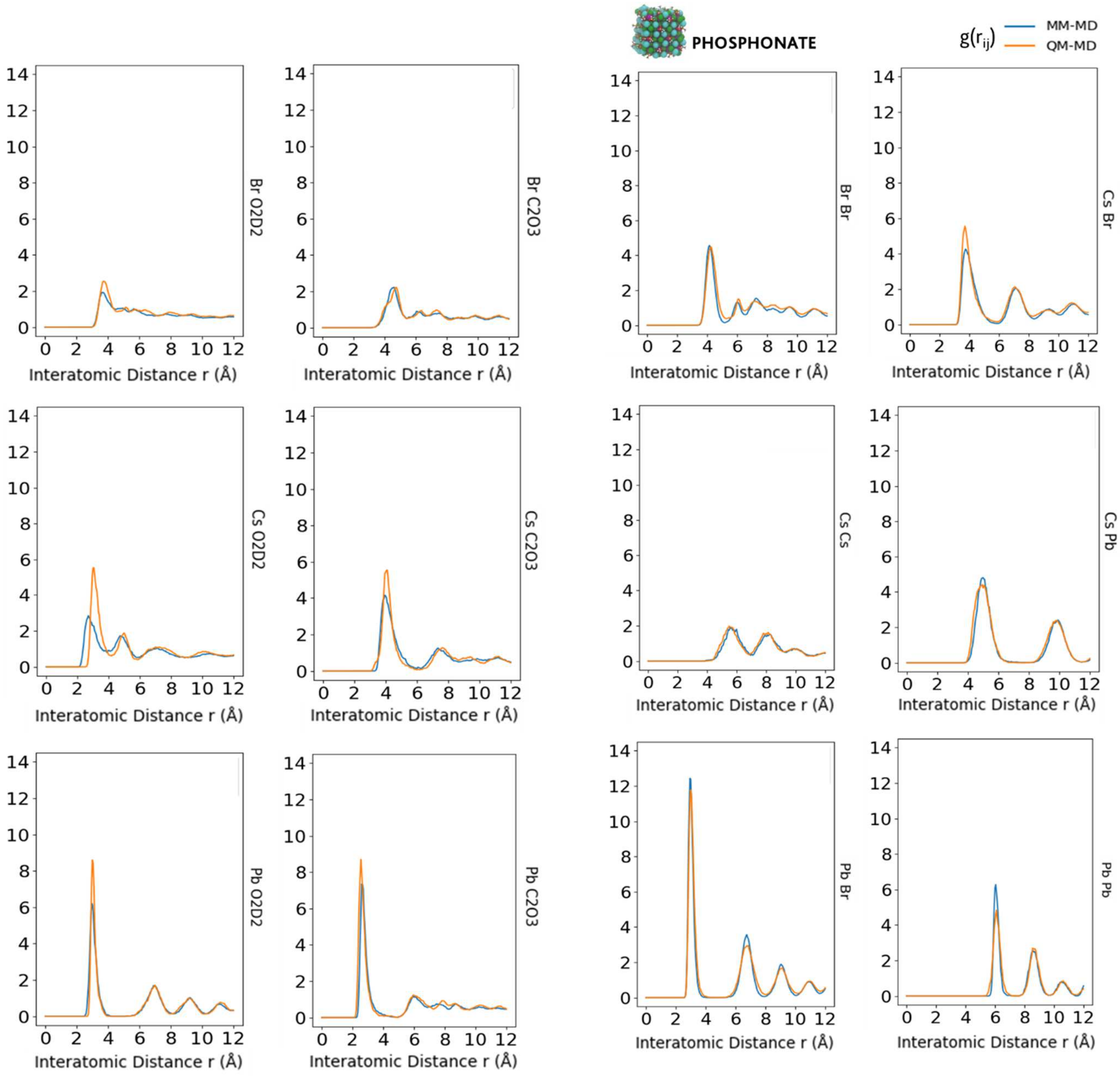
PHOSPHONATE
g(rij)
MM-MD
QM-MD
Br O2D2
Br C2O3
Br Br
Cs Br
Cs O2D2
Cs C2O3
Cs Cs
Cs Pb
Pb O2D2
Pb C2O3
Pb Br
Pb Pb
Interatomic Distance r (Å)

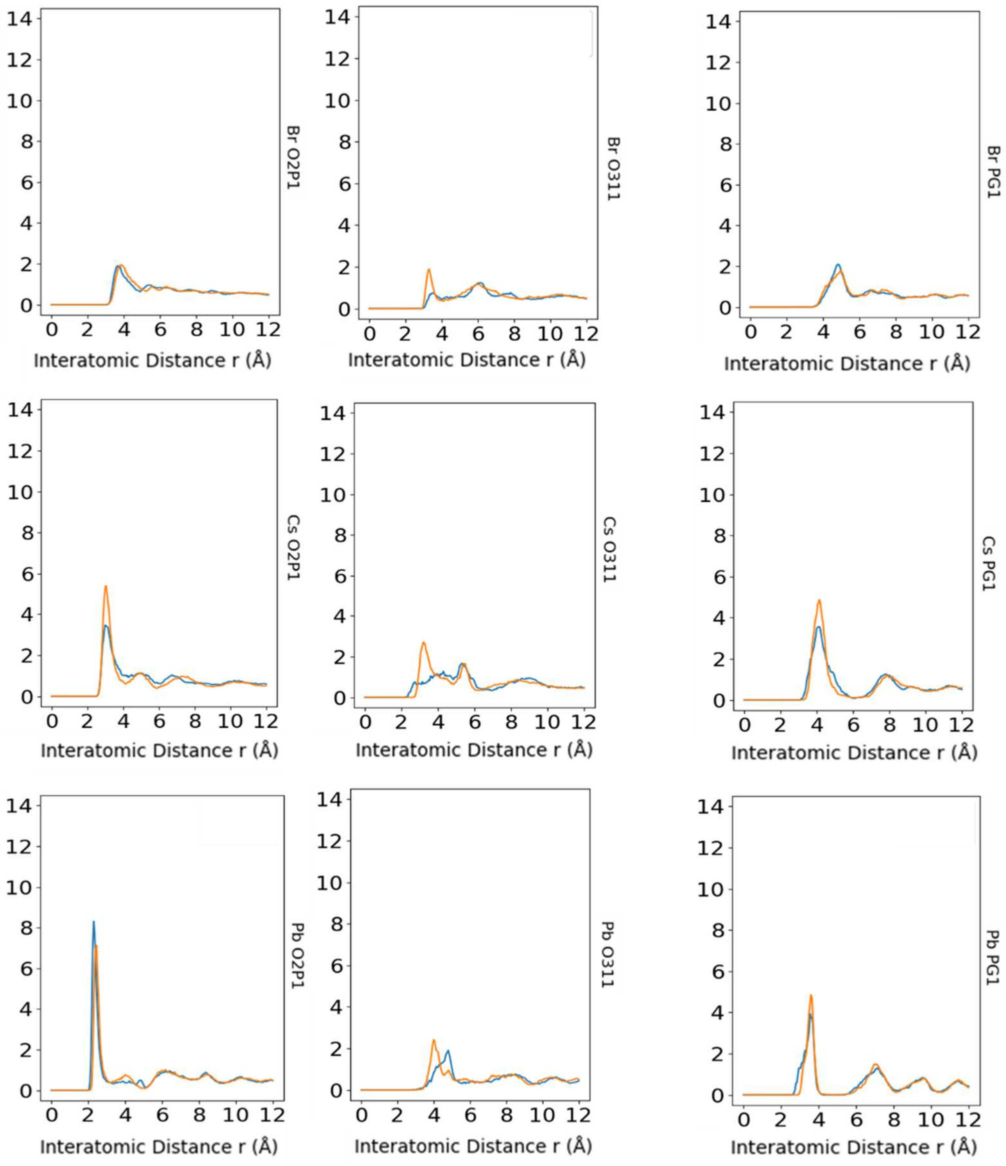
Br O2P1
Br O311
Br PG1
Cs O2P1
Cs O311
Cs PG1
Pb O2P1
Pb O311
Pb PG1
Interatomic Distance r (Å)

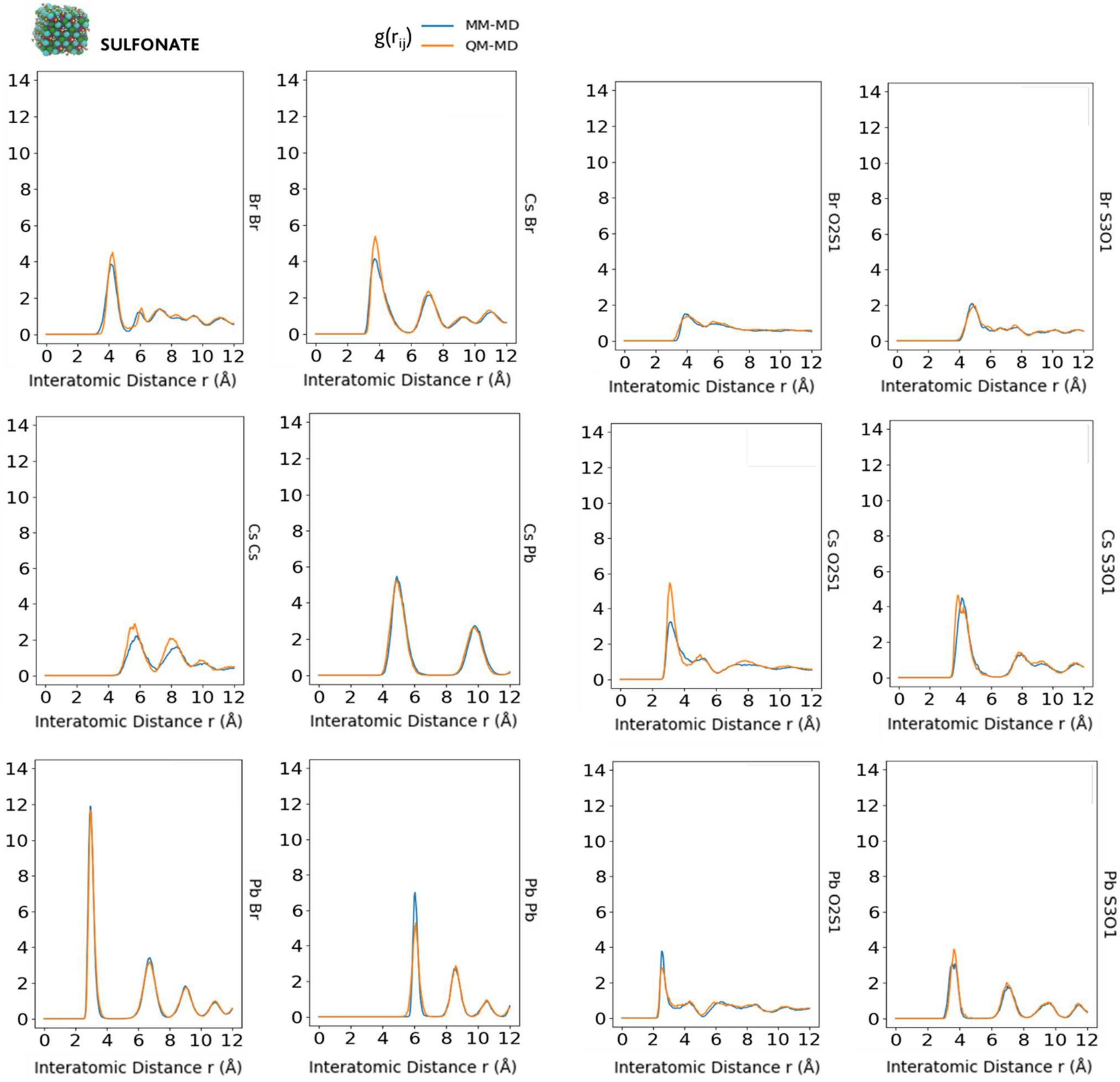
SULFONATE
g(r_ij)
MM-MD
QM-MD
Br Br
Cs Br
Br O2S1
Br S3O1
Cs Cs
Cs Pb
Cs O2S1
Cs S3O1
Pb Br
Pb Pb
Pb O2S1
Pb S3O1
Interatomic Distance r (Å)

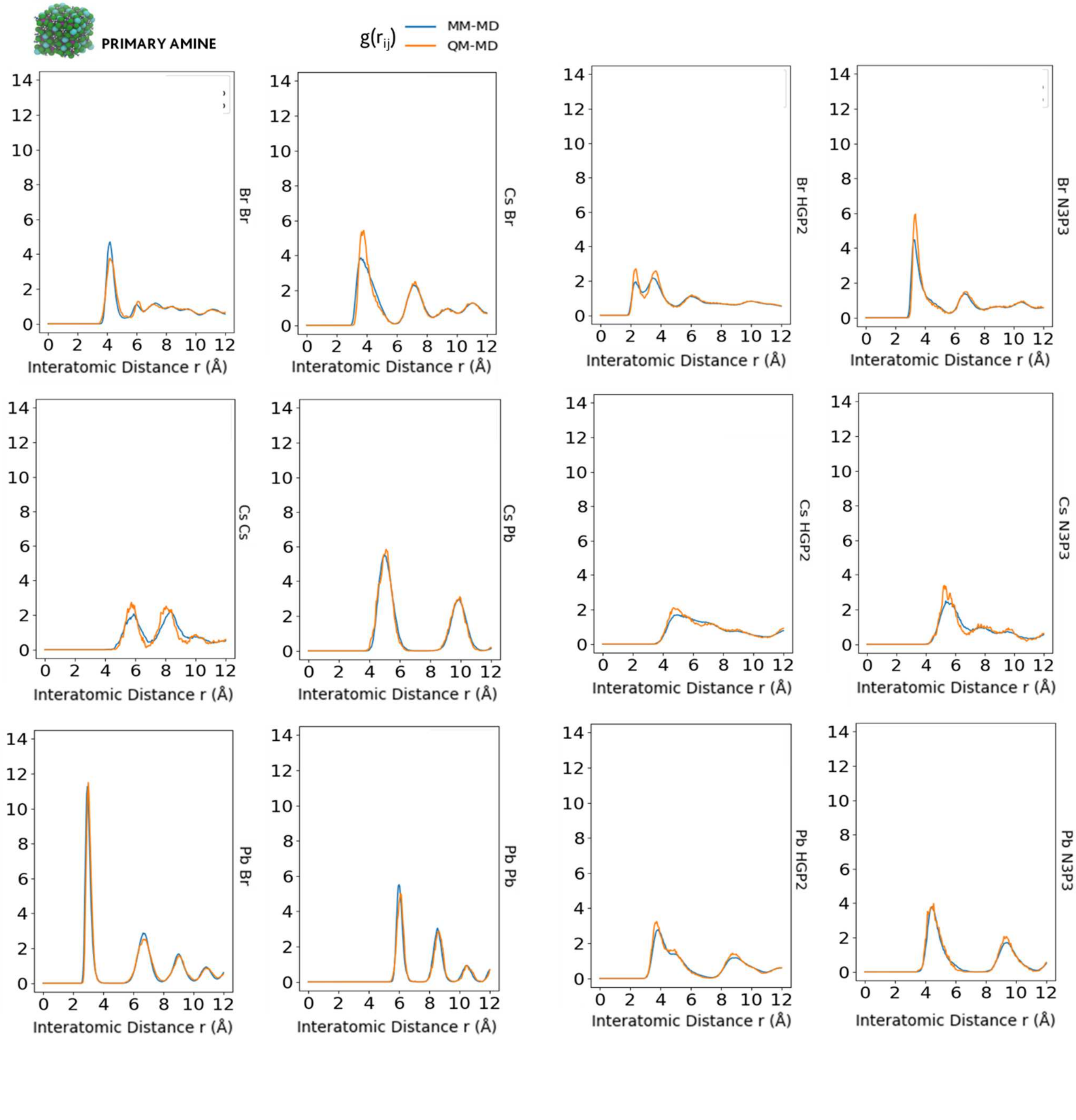
PRIMARY AMINE
g(r_ij)
MM-MD
QM-MD
Br Br
Cs Br
Br HGP2
Br N3P3
Cs Cs
Cs Pb
Cs HGP2
Cs N3P3
Pb Br
Pb Pb
Pb HGP2
Pb N3P3
Interatomic Distance r (Å)

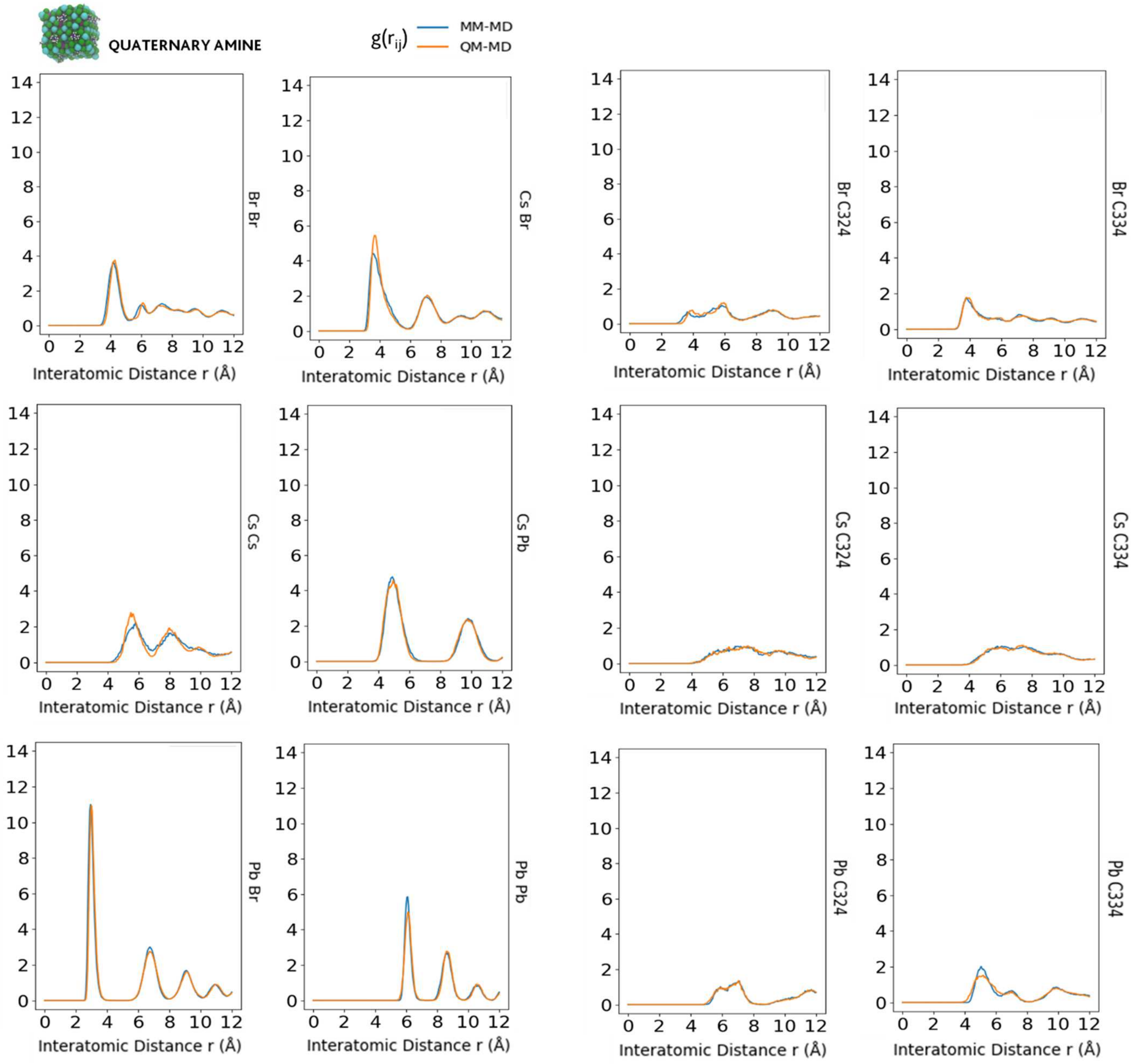
QUATERNARY AMINE
g(r_ij)
MM-MD
QM-MD
Br Br
Cs Br
Br C324
Br C334
Cs Cs
Cs Pb
Cs C324
Cs C334
Pb Br
Pb Pb
Pb C324
Pb C334
Interatomic Distance r (Å)

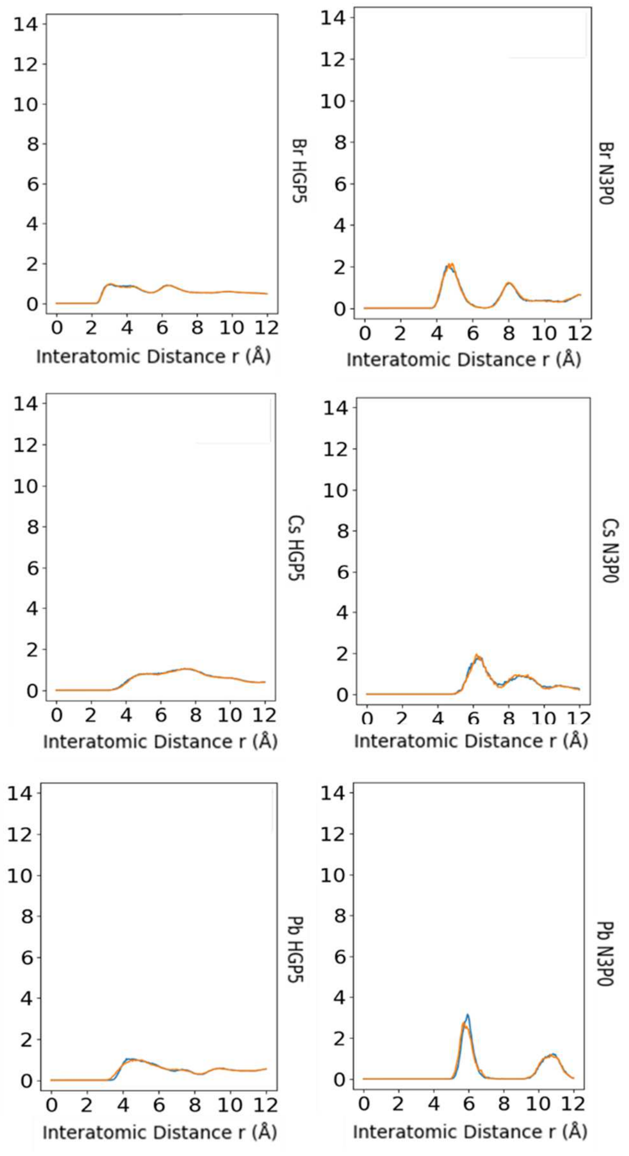

Figure S1. Comparison between ab-initio computed RDFs (QM-MD, orange plots) and ARMC-fitted RDFs (MM-MD, blue plots), computed using the optimized FF parameters, for the NC core and the ligand-capped NC models at the best iterations (i.e. set of parameters with the lowest overall error).

S2. Free energy plots

The comparison between the classically fitted free energy distributions and the quantum-mechanically computed reference plots, obtained by the application of Equation (2) to the RDF plots in Figure S1, are hereby represented for the models involved in the simulations is hereby provided in Figure S2.

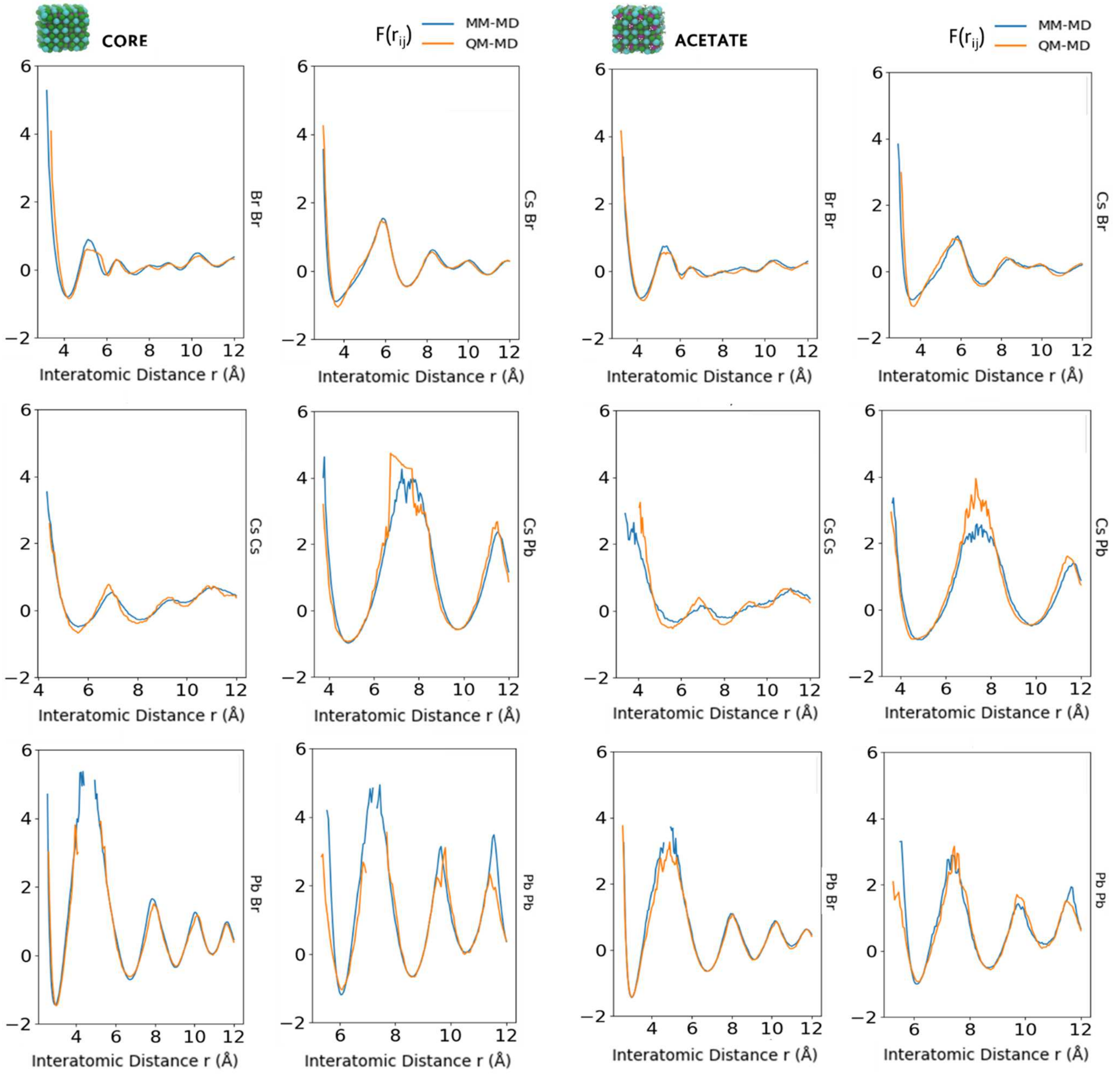

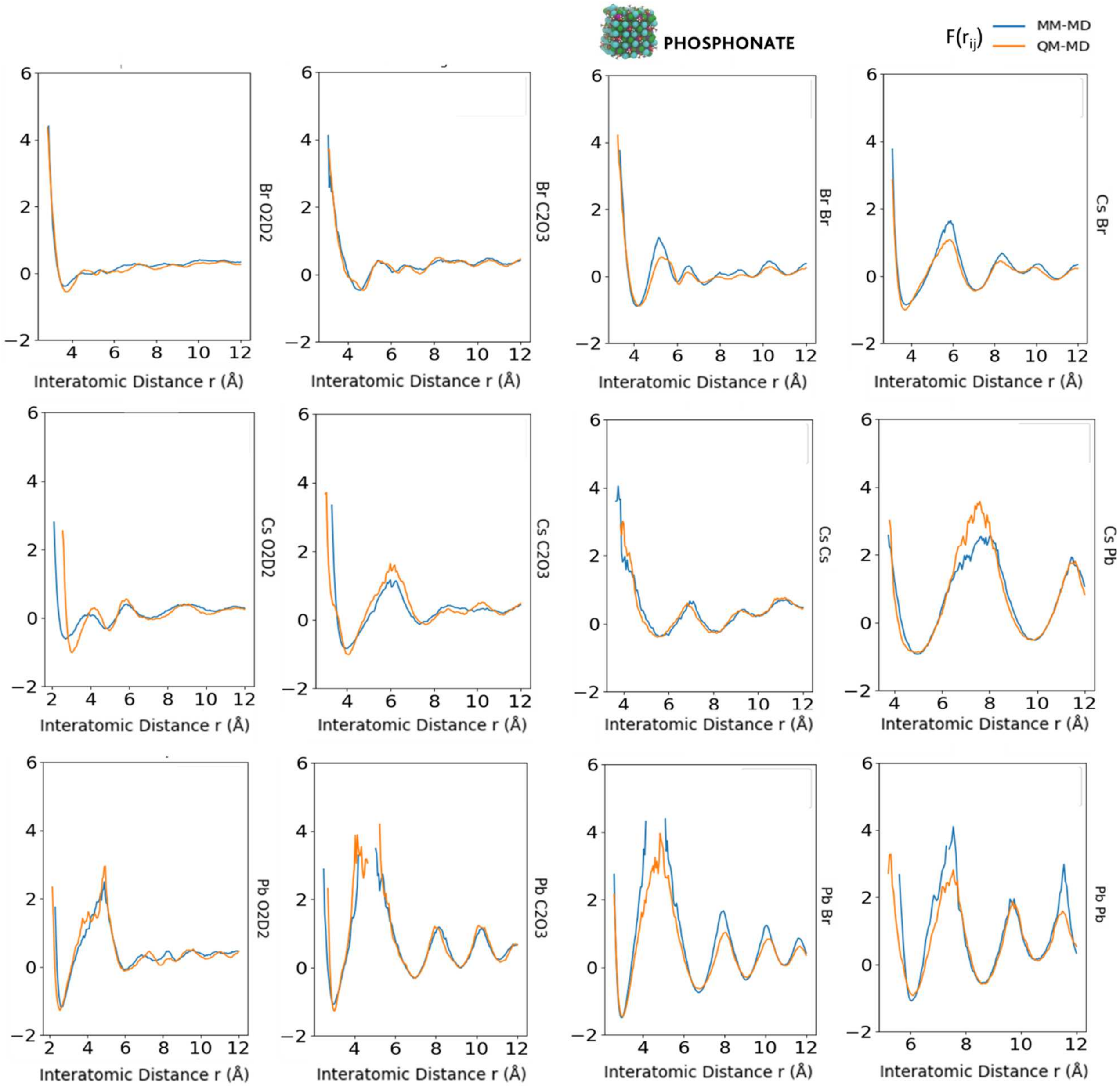
PHOSPHONATE
F(r_ij)
MM-MD
QM-MD
Br O2D2
Br C2O3
Br Br
Cs Br
Cs O2D2
Cs C2O3
Cs Cs
Cs Pb
Pb O2D2
Pb C2O3
Pb Br
Pb Pb
Interatomic Distance r (Å)

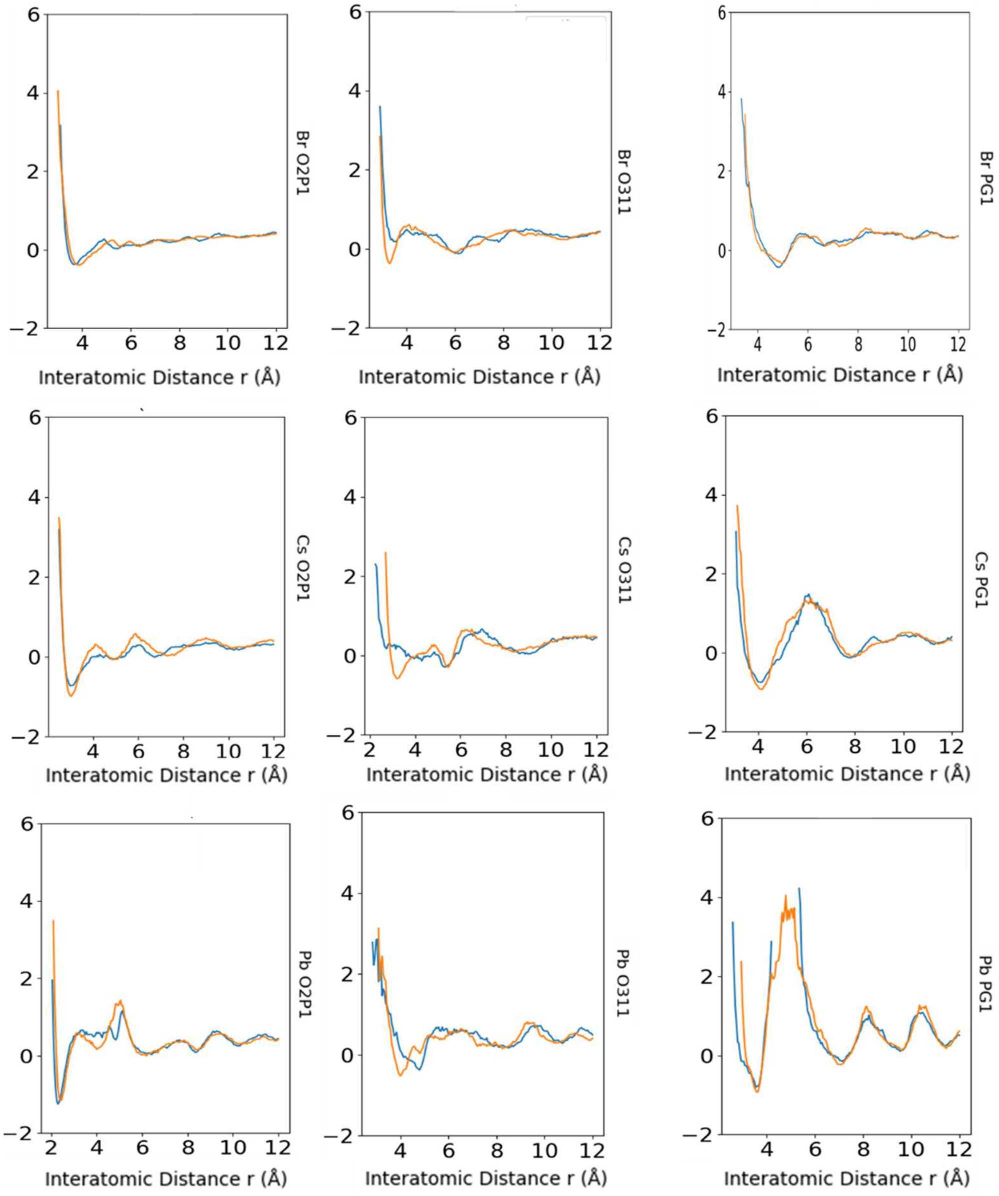
Br O2P1
Br O311
Br PG1
Cs O2P1
Cs O311
Cs PG1
Pb O2P1
Pb O311
Pb PG1
Interatomic Distance r (Å)

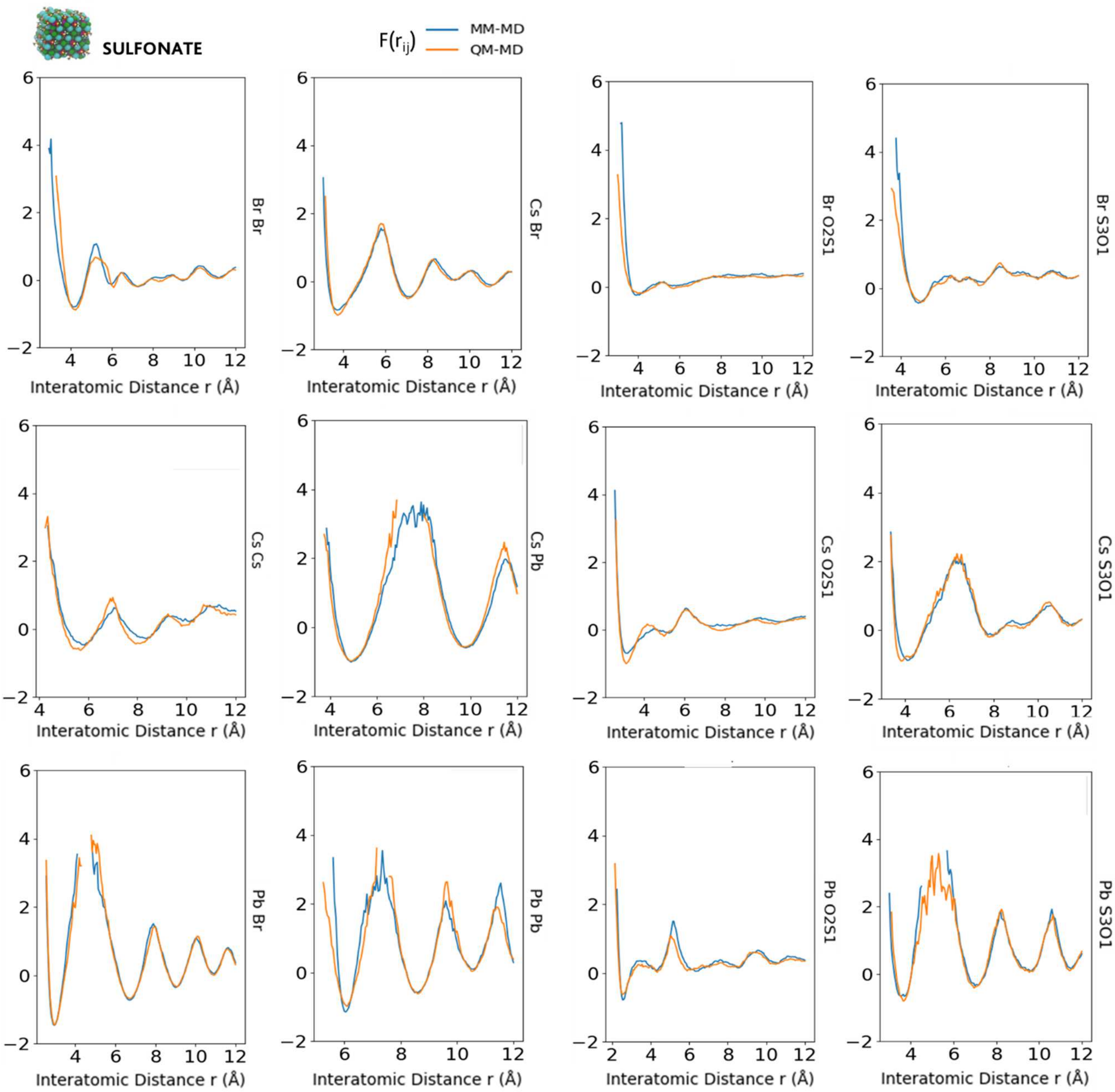

SULFONATE
F(r_ij)
MM-MD
QM-MD
Interatomic Distance r (Å)
Br Br
Cs Br
Br O2S1
Br S3O1
Cs Cs
Cs Pb
Cs O2S1
Cs S3O1
Pb Br
Pb Pb
Pb O2S1
Pb S3O1

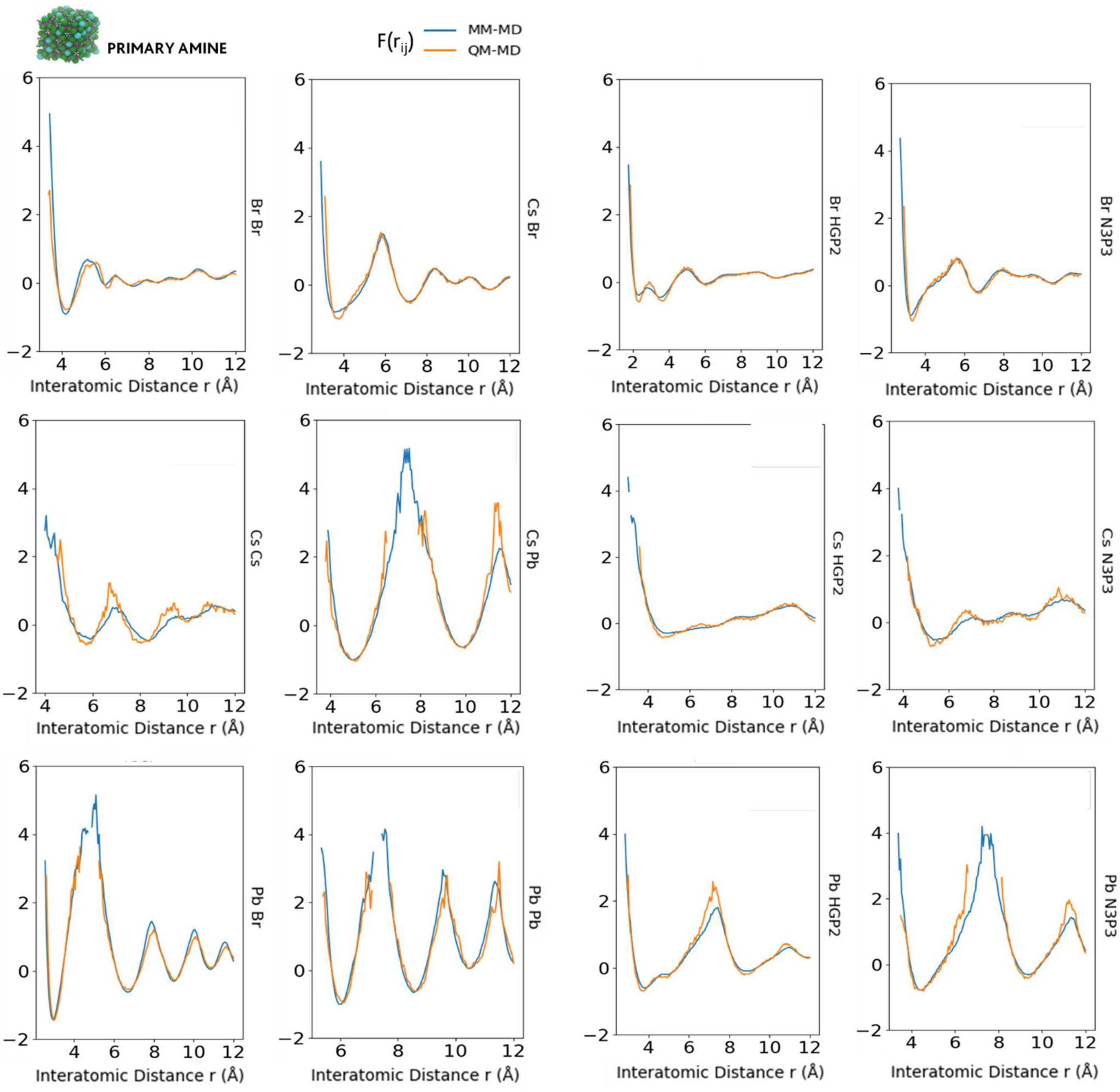

PRIMARY AMINE
F(r_ij)
MM-MD
QM-MD
Br Br
Cs Br
Br HGP2
Br N3P3
Cs Cs
Cs Pb
Cs HGP2
Cs N3P3
Pb Br
Pb Pb
Pb HGP2
Pb N3P3
Interatomic Distance r (Å)

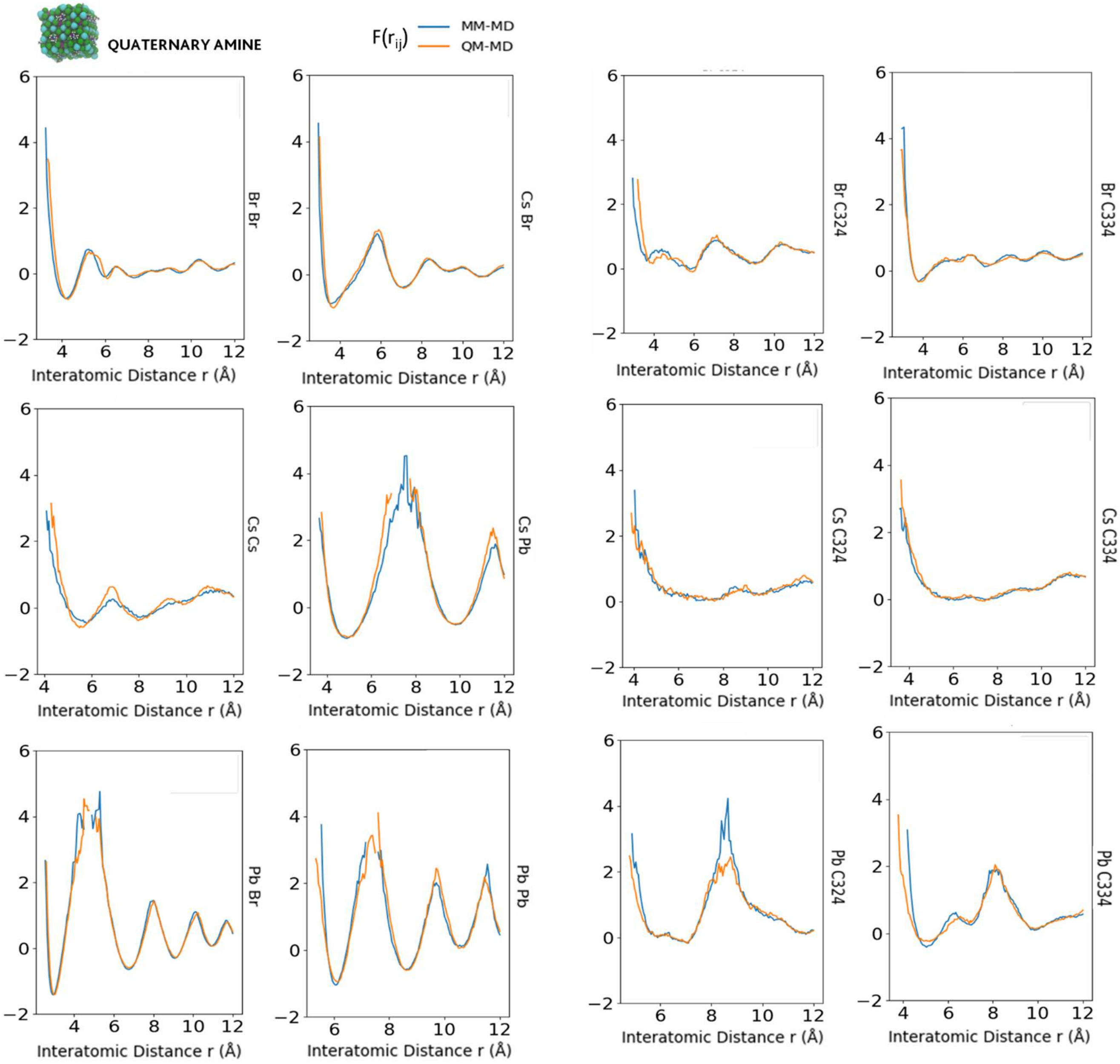
QUATERNARY AMINE
F(r_ij)
MM-MD
QM-MD
Br Br
Cs Br
Br C324
Br C334
Cs Cs
Cs Pb
Cs C324
Cs C334
Pb Br
Pb Pb
Pb C324
Pb C334
Interatomic Distance r (Å)

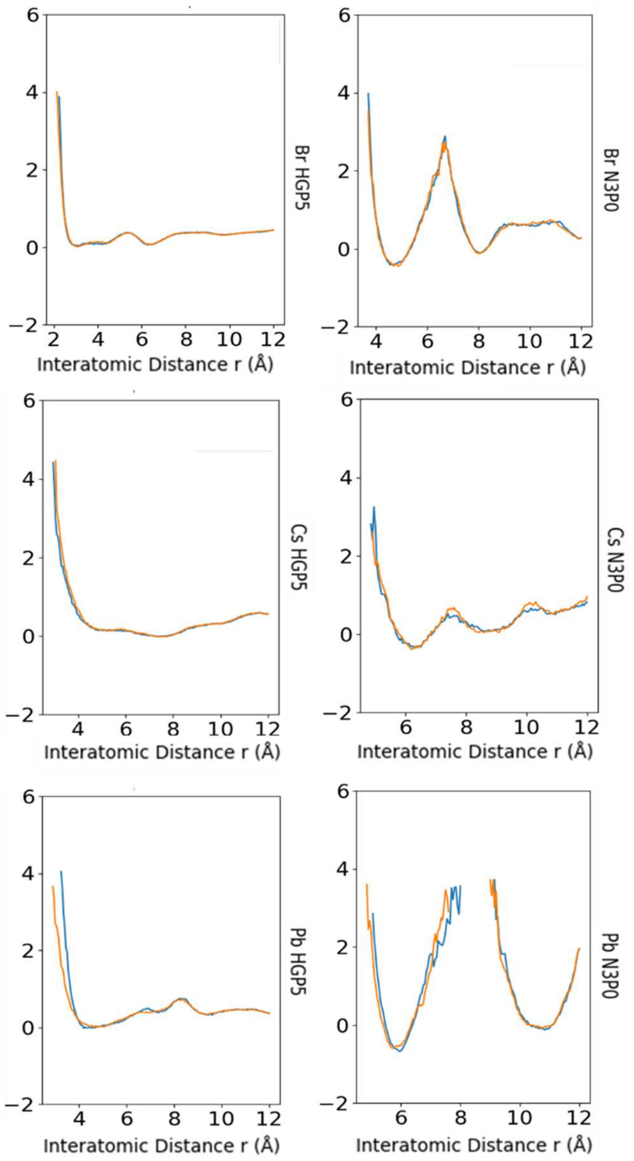

Figure S2. Comparison between QM-computed free energy plots (in orange) and MM-based free energy plots (in blue) for the NC core and the ligand-capped NC models at the best iterations (i.e. set of parameters with the lowest overall error).

S3. CHARMM FF tail parameters

The CHARMM FF[3] parameters (charges *q* and LJ interatomic distances *σ)* obtained for the atoms of the ligand tails are hereby represented in Table 1 for both the NC core and the ligand-capped NC models.

Table S3. Calculated Force Field Frozen Parameters for Ligand-Capped $CsPbBr_3$ NC: Charge (e) and Sigma (nm)[a]

Acetate		Phosphonate		Sulfonate		Primary amine		Quaternary amine	
Ligand charges									
C331	-0.3700	HGP1	0.3600	C331	-0.0770	C334	-0.3700	C321	-0.1800
HGA3	0.0900	C331	-0.7400	HGA3	0.0900	HGA3	0.0900	C331	-0.2700
		HGA3	0.0900					HGA3	0.0900
								HGA2	0.0900
Sigma Ligand-core (nm)									
Cs C331	0.384	Cs HGP1	0.261	Cs C331	0.384	Cs C334	0.384	Cs C321	0.380
Pb C331	0.374	Pb HGP1	0.281	Pb C331	0.374	Pb C334	0.389	Pb C321	0.370
Br C331	0.369	Br HGP1	0.228	Br C331	0.369	Br C334	0.369	Br C321	0.366
Cs HGA3	0.321	Cs C331	0.383	Cs HGA3	0.321	Cs HGA3	0.321	Cs C331	0.384
Pb HGA3	0.311	Pb C331	0.374	Pb HGA3	0.311	Pb HGA3	0.311	Pb C331	0.374
Br HGA3	0.306	Br C331	0.369	Br HGA3	0.306	Br HGA3	0.306	Br C331	0.369
		Cs HGA3	0.321					Cs HGA3	0.321
		Pb HGA3	0.311					Pb HGA3	0.311
		Br HGA3	0.306					Br HGA3	0.306
								Cs HGA2	0.321
								Pb HGA2	0.311
								Br HGA2	0.306

[a] Their units of measurement are the default ones employed by the CP2K package[4] in MD simulations: the charges (q) are provided in elementary charge units, while the distances (σ) are reported in nm.

S4. Fitted charges

The trend shown by the charges over the course of the fitting procedure is hereby represented for the six models involved in the simulations in Figure S4.

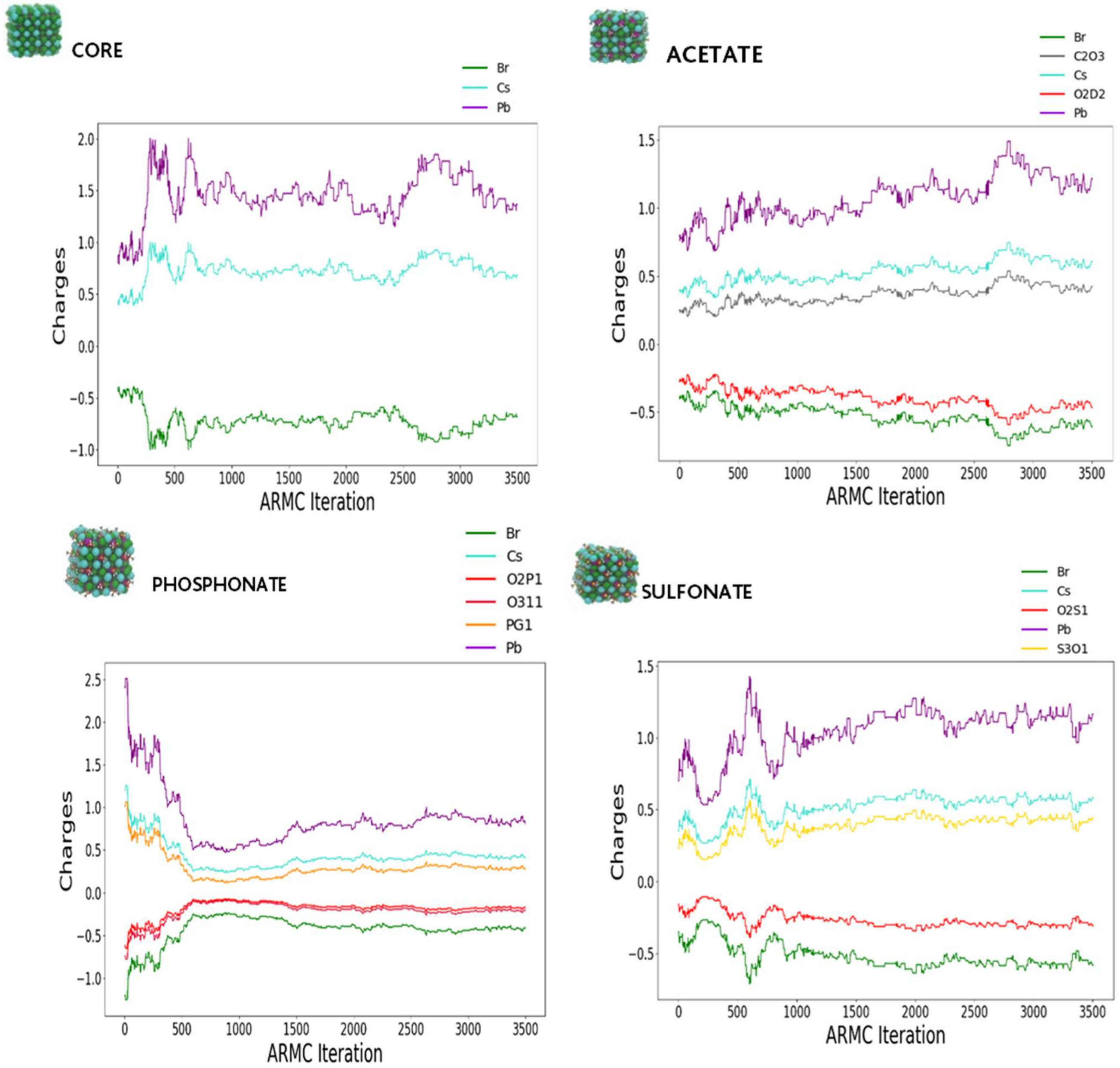

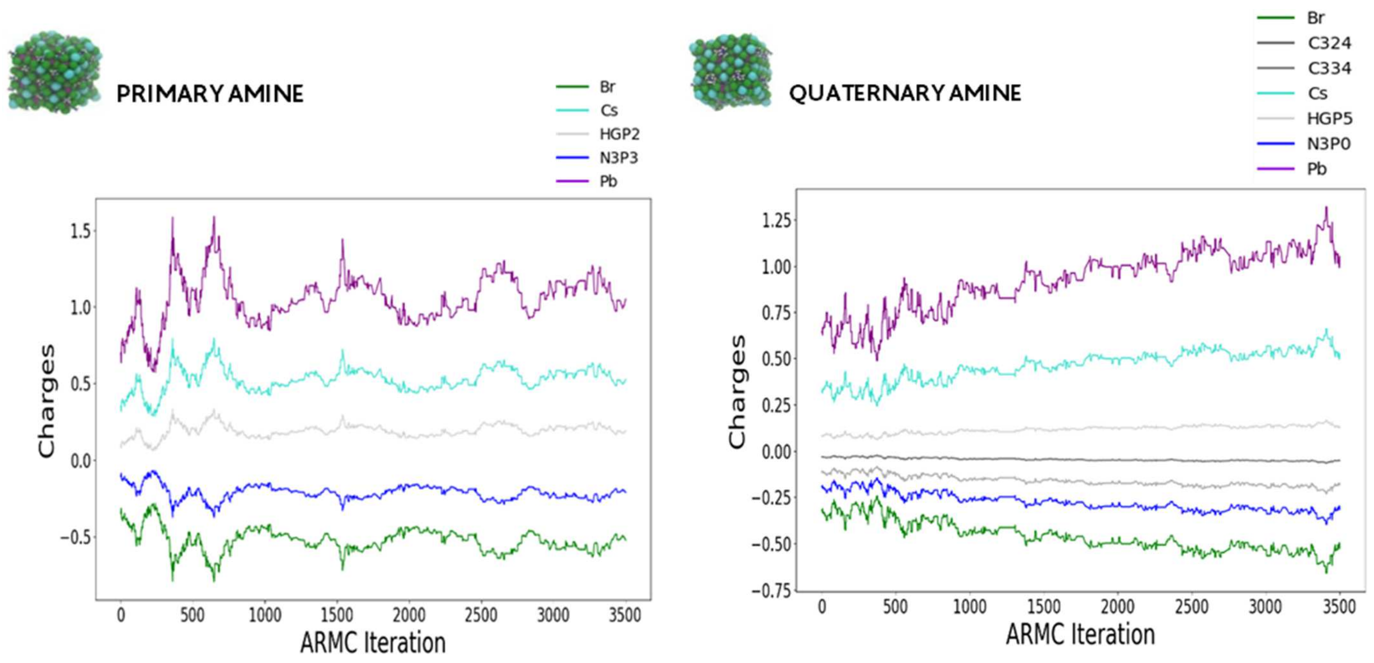

Figure S4. Representation of the trend shown by the charges over the course of the ARMC parametrization procedure for the six analyzed NC models.

S5. Fitted sigmas

The trend shown by the LJ distances σ over the course of the fitting procedure is hereby represented for the six models involved in the simulations in Figure S5.

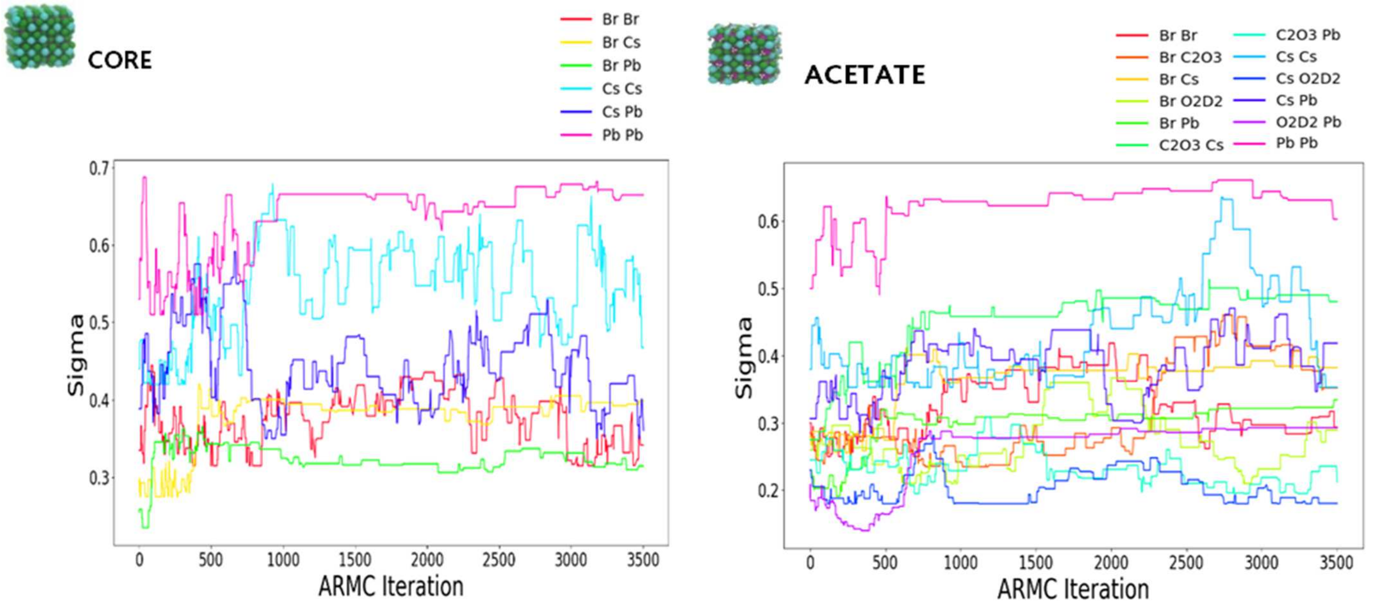

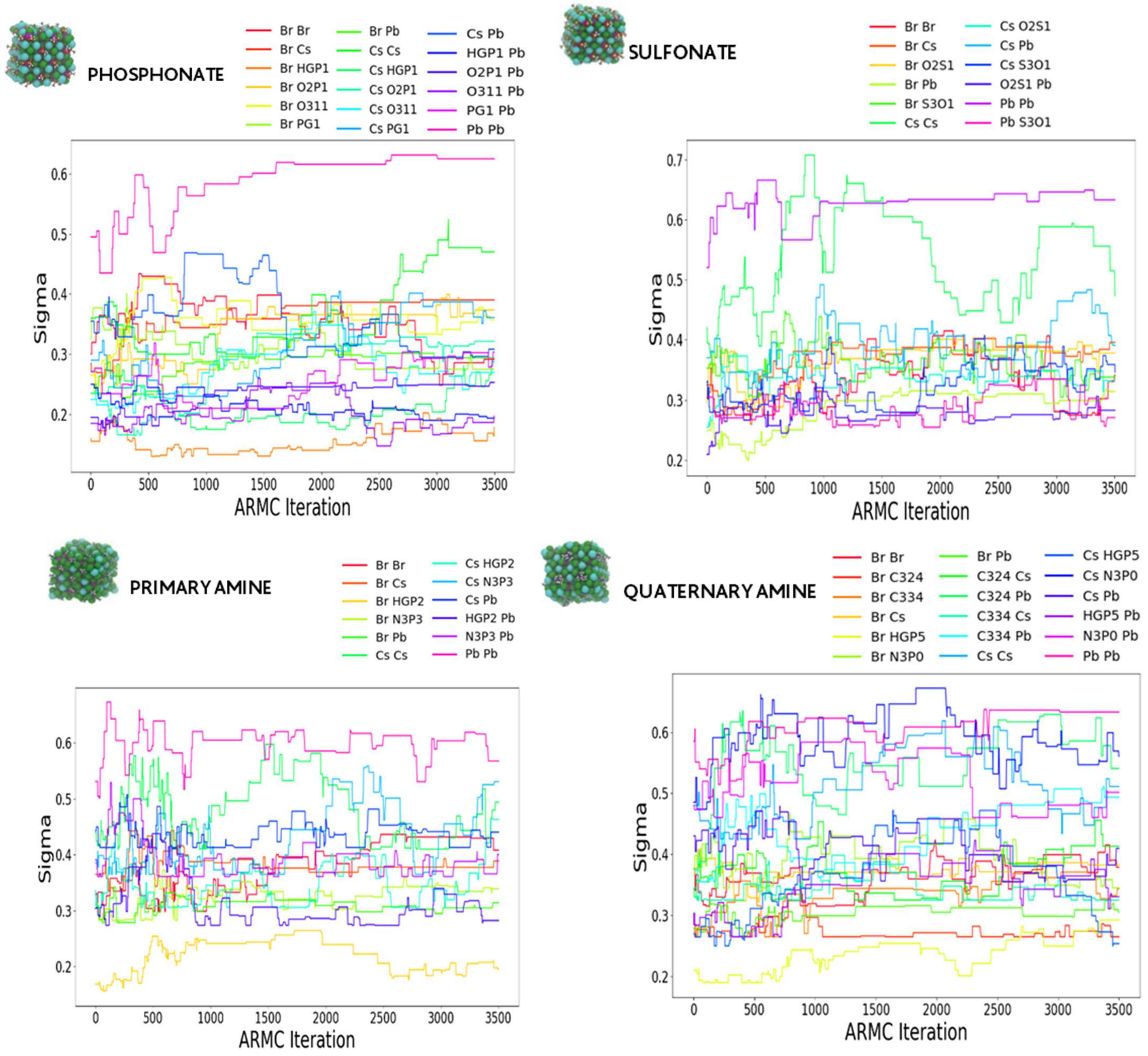

Figure S5. Representation of the trend shown by the LJ distances over the course of the ARMC parametrization procedure for the six analyzed NC models.

.

S6. Vibrational densities of states (vDOS)

The comparison between the classically fitted vibrational densities of states (vDOS) and the quantum-mechanically computed reference plots are hereby represented for the models involved in the simulations is hereby provided in Figure S6.

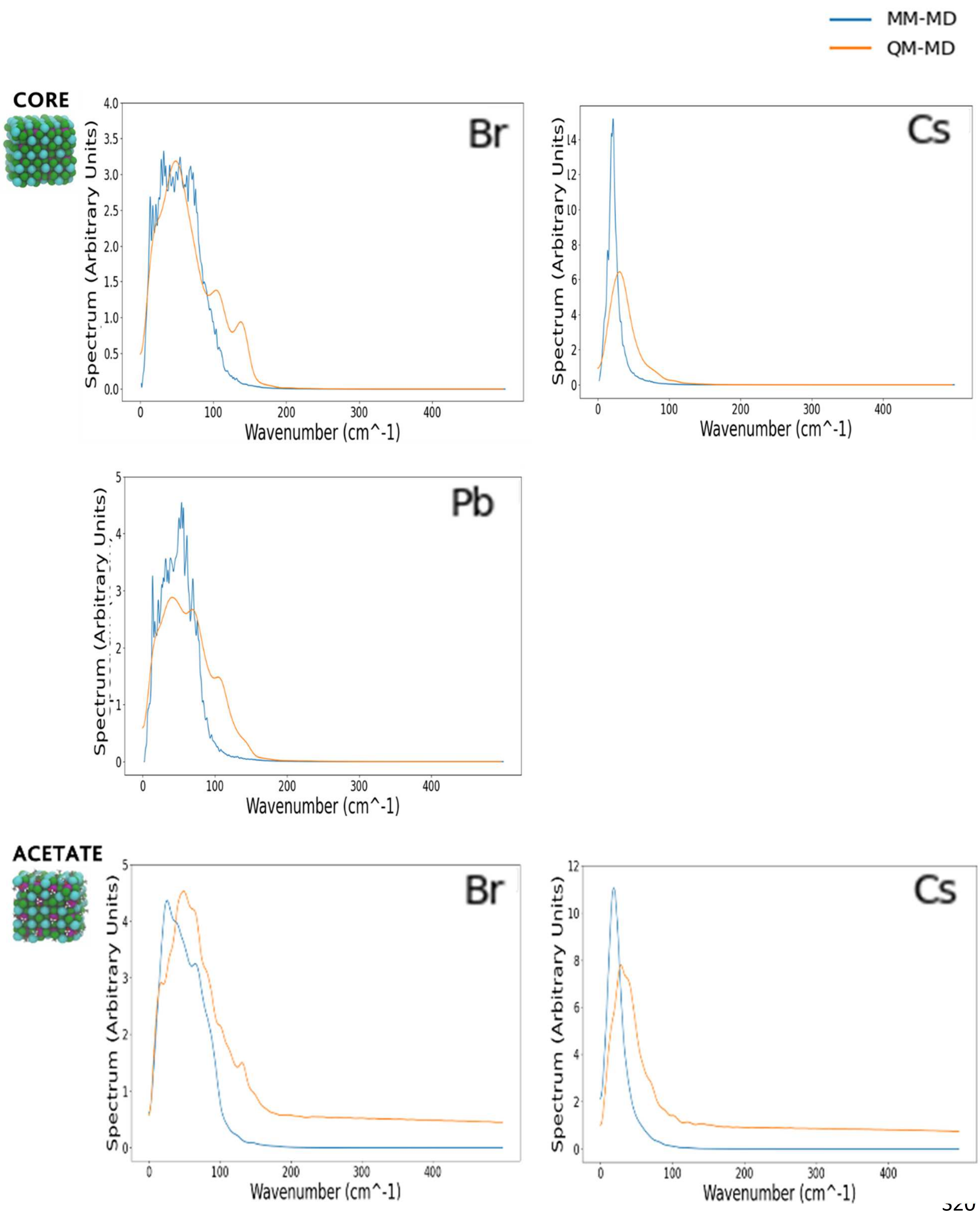

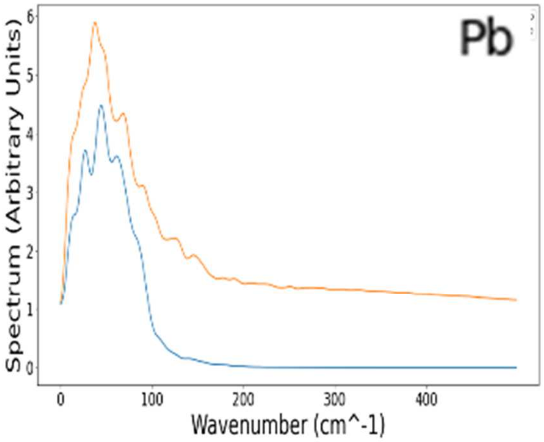

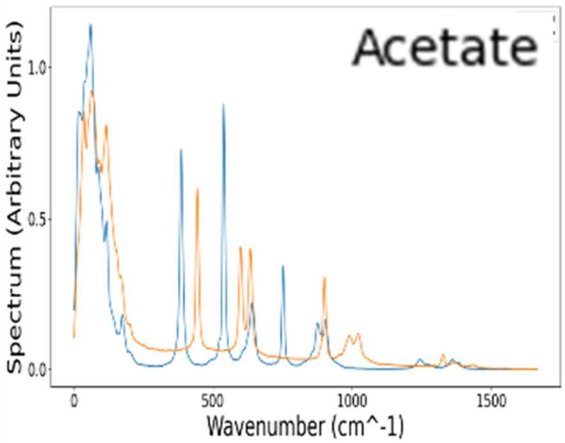

PHOSPHONATE

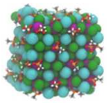

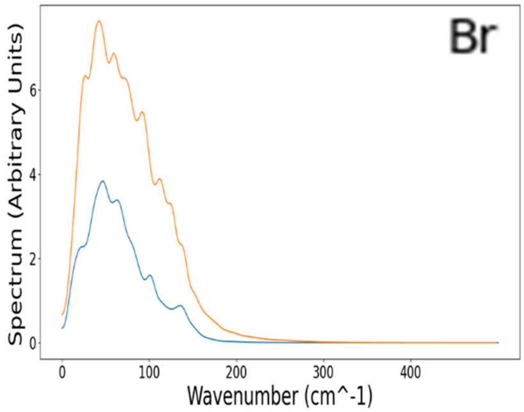

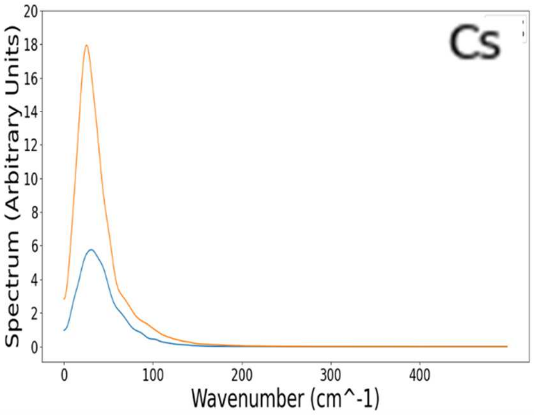

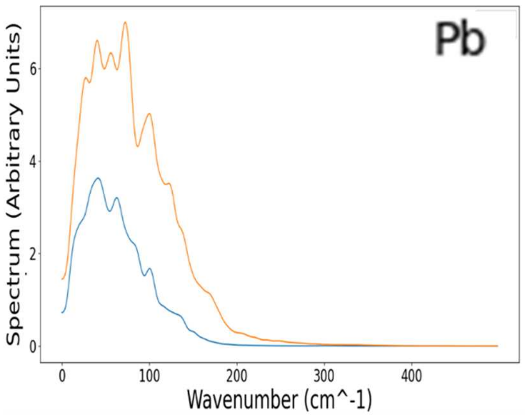

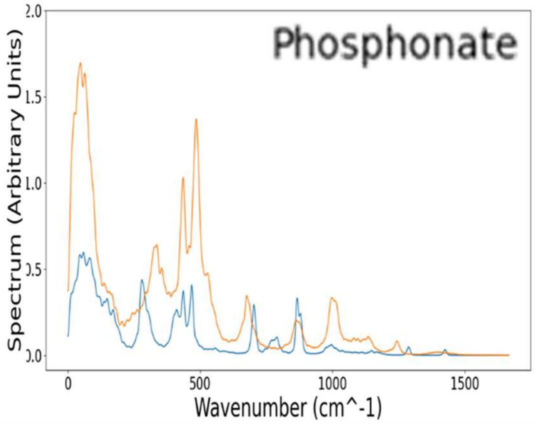

SULFONATE

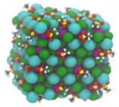

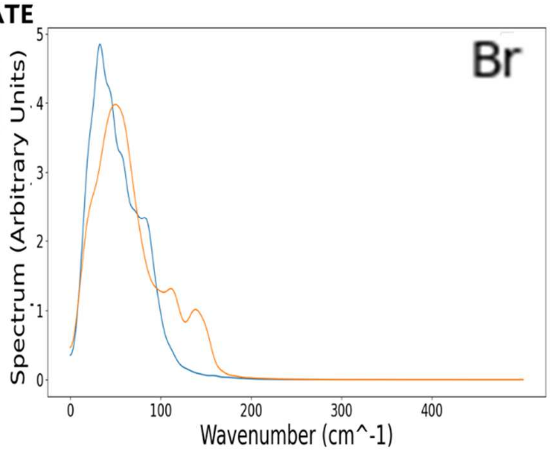

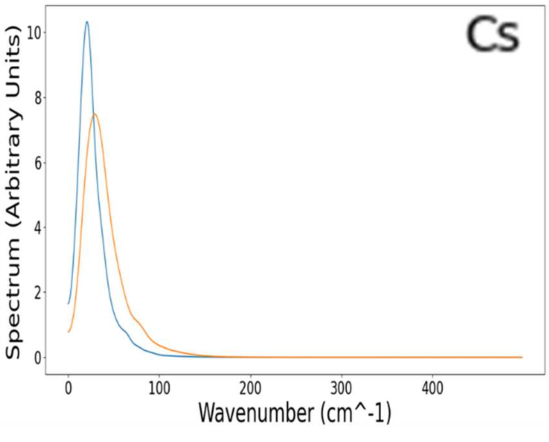

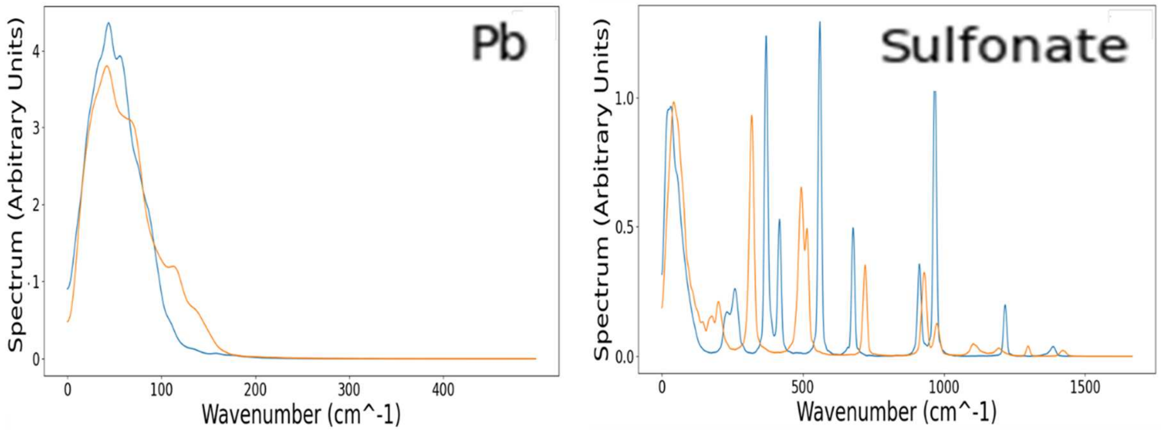

PRIMARY AMINE

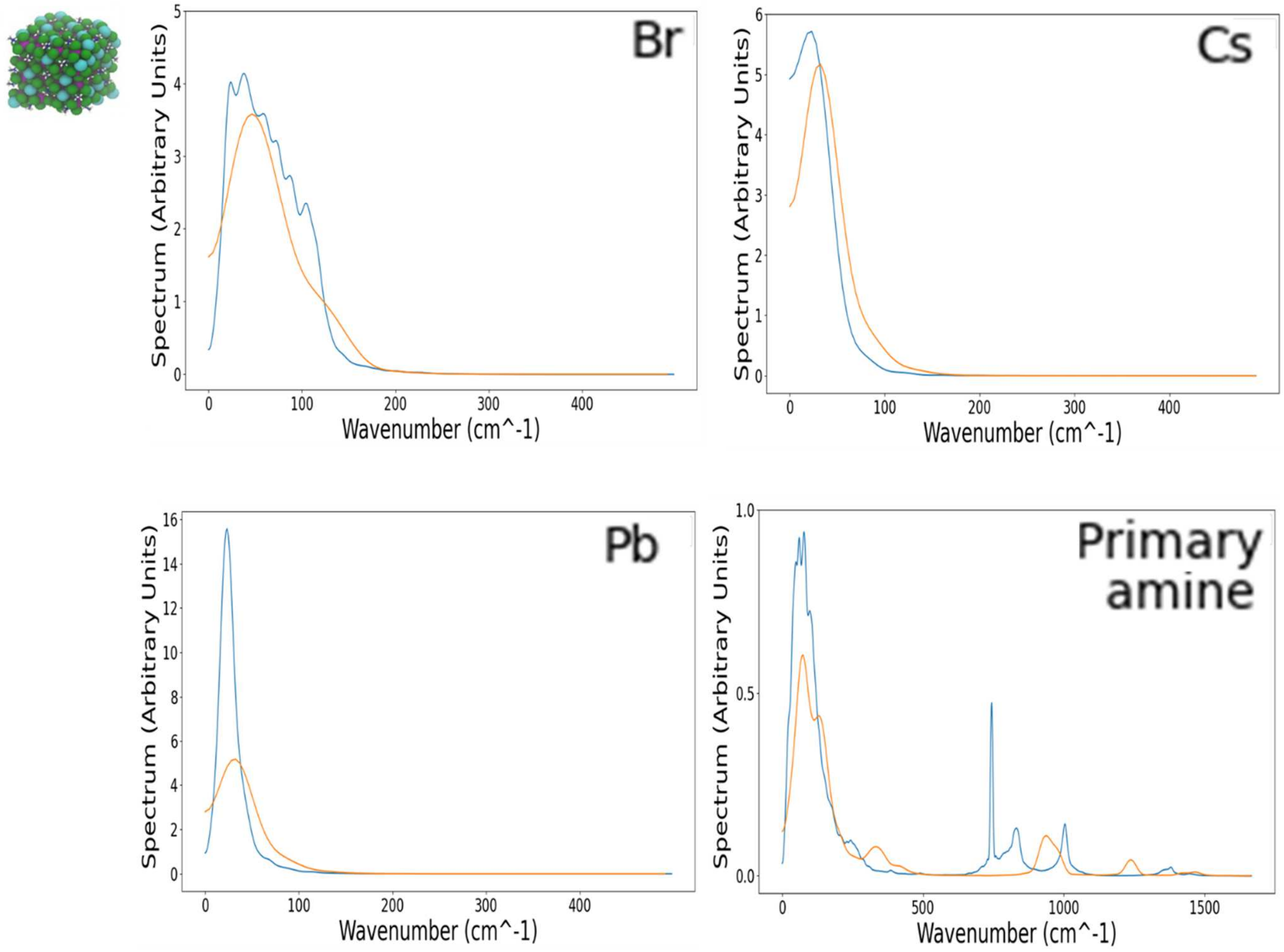

QUATERNARY AMINE

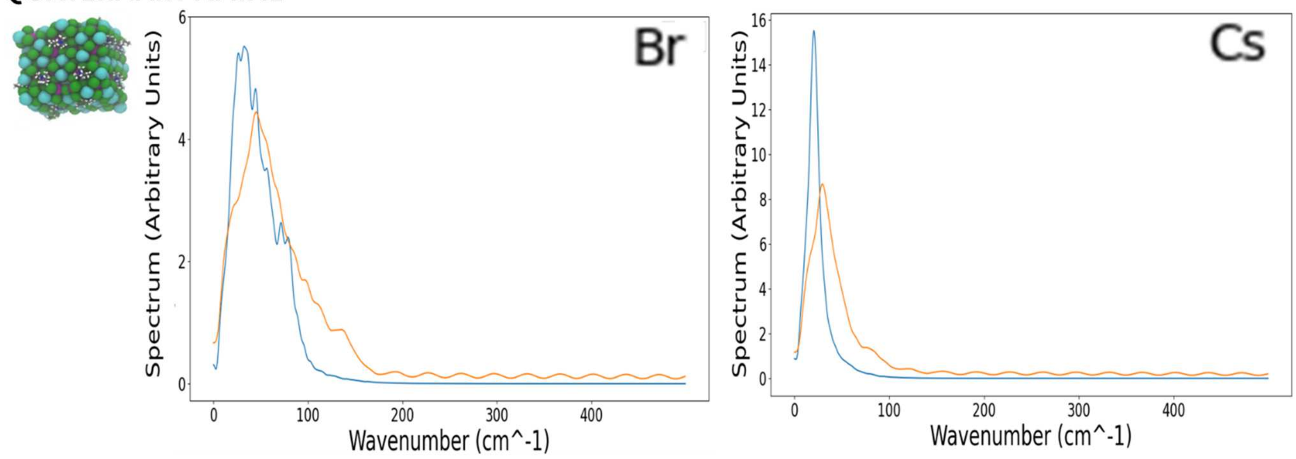

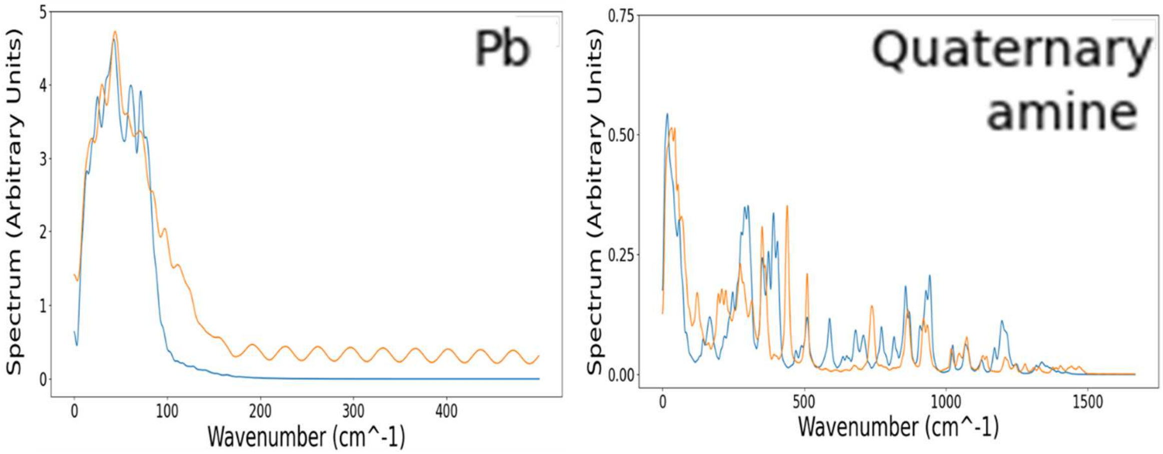

Figure S6. Representation of the vDOS plots obtained for the six optimized NC models

S7. Total energy plots

The plots containing the average total energies (i.e. the sum of the kinetic and potential energies) of the MM-MD simulations obtained for the upscaled 5.0 nm NC core and for the 5 real size ligand-capped models involved in the simulations are provided in Figure S7.

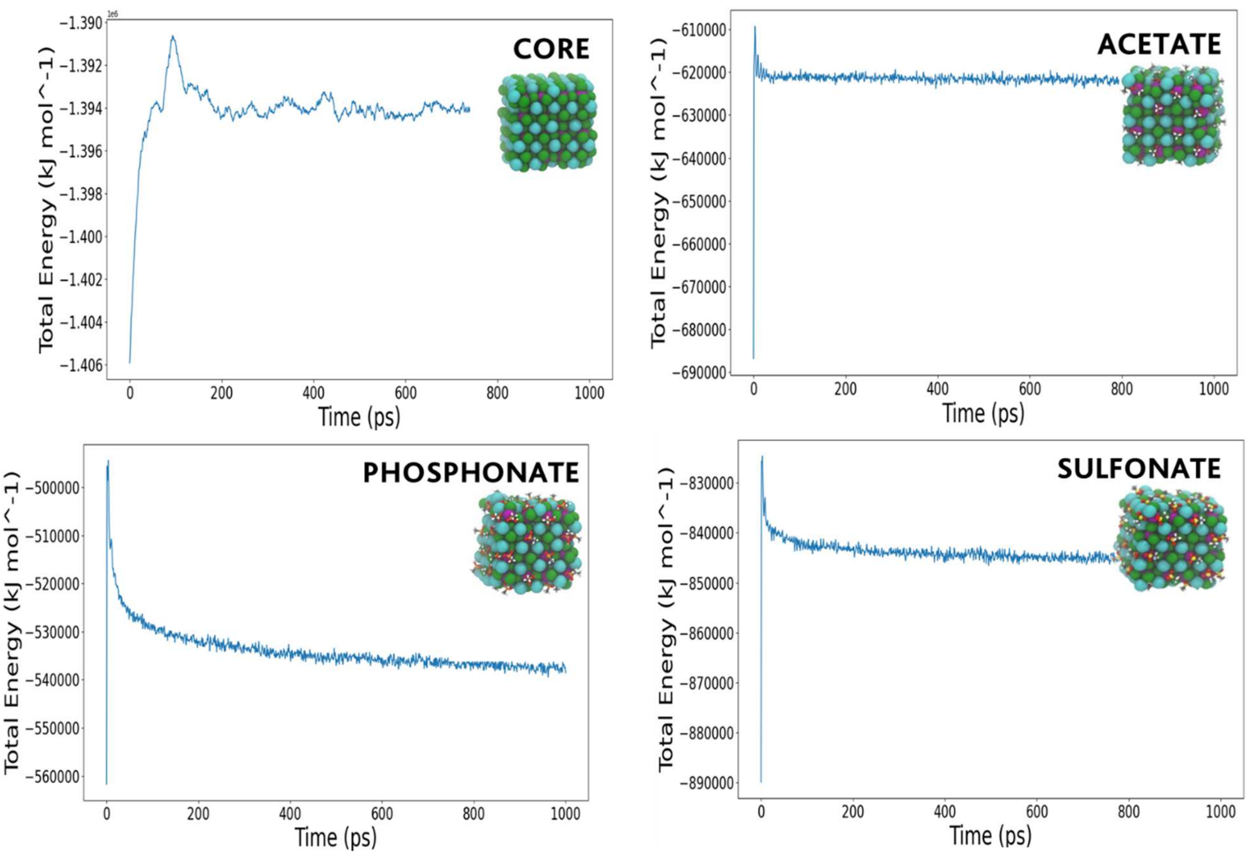

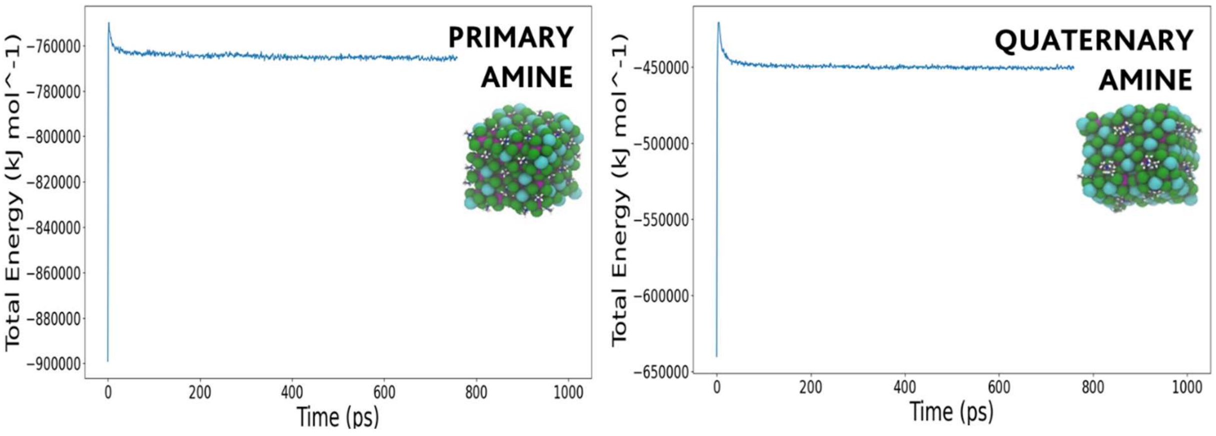

Figure S7. Free energy plots obtained procedure for the NC core and for the five ligand-capped models.

S8. Binding energies

The binding energies $\Delta E_{binding}$ between the organic ligands and the NC models, defined as the difference between the energies of the bound species (the NC models, E_{NC} in the used notation) and the unbound species (the ligand-ion pairs, denoted as E_{lig}, and the NC model without that pair, E_{NC-lig}), can be schematized as:

$$\Delta E_{binding} = E_{NC} - (E_{lig} + E_{NC-lig})$$

The results which have been obtained in these calculations, comparing between the DFT- and MM-based binding energies, are displayed in **Table S8**.

Table S8. Calculated QM and MM Binding Energies for Ligand-Capped $CsPbBr_3$ NC.

Binding energy (kcal mol^{-1})				
***Ab-initio* (QM) calculation**				
**Acetate**	**Phosphonate**	**Sulfonate**	**Primary amine**	**Quaternary amine**
-44.1	-48.2	-53.1	-43.3	-32
**FF-based (MM) calculation**				
**Acetate**	**Phosphonate**	**Sulfonate**	**Primary amine**	**Quaternary amine**
-32.9	-74.6	-33.8	-42.6	-22.8

The trends shown by this table can be motivated by the fact that the fitting procedure has been executed using the RDFs of the NC model as a reference. These RDFs don't provide an accurate depiction of the ligand-ion couples. The flexibility of our model would us to investigating the binding energies of these models by performing a global fit using both the NC model and the ligand-ion couples in vacuum, but this attempt goes beyond the purpose of this work.

S9. Example of yaml input script – single ARMC optimization

An example of the yaml input script of auto-FOX used for the FF fitting of the $CsPbBr_3$ NC capped with acetate is provided in the following section.

```
param:
  charge:
    param: charge
```

```
        Cs: 0.4
        Pb: 0.8
        Br: -0.4
        C2O3: 0.25
        O2D2: -0.275
        constraints:
            - '0 < Cs < 1.5'
            - '0 < Pb < 2'
            - '-1.5 < Br < 0'
            - 'Pb == -2 * $LIGAND == -2 * Br == 2 * Cs'

    lennard_jones:
        - param: epsilon
          unit: kjmol
          frozen:
              guess: uff
        - param: sigma
          unit: nm
          Cs Cs:  0.553
          Cs Pb:  0.367
          Br Cs:  0.363
          Pb Pb:  0.610
          Br Pb:  0.298
          Br Br:  0.379
          C2O3 Cs: 0.437
          C2O3 Pb: 0.238
          Br C2O3: 0.233
          Cs O2D2: 0.201
          O2D2 Pb: 0.264
          Br O2D2: 0.330
          constraints:
              - 'Cs Cs   > 0.523'
              - 'Cs Pb   > 0.337'
              - 'Br Cs   > 0.333'
              - 'Pb Pb   > 0.580'
              - 'Br Pb   > 0.268'
              - 'Br Br   > 0.349'
              - 'C2O3 Cs > 0.407'
              - 'C2O3 Pb > 0.218'
              - 'Br C2O3 > 0.213'
              - 'Cs O2D2 > 0.181'
              - 'O2D2 Pb > 0.234'
              - 'Br O2D2 > 0.310'
          frozen:
              guess: uff

psf:
    rtf_file: acetate.rtf
    ligand_atoms: [C, O, H]

pes:
    rdf:
        func: FOX.MultiMolecule.init_rdf
        err_func: FOX.armc.err_normalized_weighted
        kwargs:
            atom_pairs:
              - [Cs, Cs]
              - [Cs, Pb]
```

```
            - [Br, Cs]
            - [Pb, Pb]
            - [Br, Pb]
            - [Br, Br]
            - [C2O3, Cs]
            - [C2O3, Pb]
            - [Br, C2O3]
            - [Cs, O2D2]
            - [O2D2, Pb]
            - [Br, O2D2]

job:
    molecule: last5000.xyz
   md_settings:
       template: qmflows.templates.md.specific.cp2k_mm
       settings:
           prm: acetate.prm
           input:
               global:
                   print_level: LOW
               force_eval:
                   mm:
                     poisson:
                        periodic: xyz
                        ewald:
                          ewald_type: spme
                          gmax: '62 62 62'
                          o_spline: 4
                   subsys:
                       cell:
                           abc: '[angstrom] 100.0 100.0 100.0'
                           periodic: xyz
               motion:
                   print:
                       cell:
                          each:
                             md: 10
                       restart:
                          each:
                             md: 10
                       trajectory:
                          each:
                             md: 10
                       velocities:
                          each:
                             md: 10
                       forces:
                          each:
                             md: 10
                   md:
                       ensemble: NVT
                       temperature: 300.0
                       timestep: 1
                       steps: 10000
                       thermostat:
                           type: csvr
                           csvr:
                               timecon: 10000
```

```
            print:
                energy:
                    each:
                        md: 10
            barostat:
                pressure: 1.01
                timecon: 10000

monte_carlo:
    logfile: armc.log
    hdf5_file: armc.hdf5
    path: ./
    folder: MM_MD_workdir
    keep_files: True
```

S10. Simultaneous ARMC optimization

An example of the yaml input script of auto-FOX used for the simultaneous FF fitting of the $CsPbBr_3$ NC capped with acetate and methylammonium is provided in the following section.

```
param:
    charge:
        param: charge
        Cs: 0.56025
        Pb: 1.1205
        Br: -0.56025
        C2O3: 0.3605 #charge of rest: -0.1. Total must be charge(Cl)
        O2D2: -0.41036 # 2xO2D2 + C2O3 + rest = Cl
        N3P3: -0.2377
        HGP2: 0.209318
        constraints:
            - '0 < Cs < 1.5'
            - '0 < Pb < 2'
            - '-1.5 < Br < 0'
            - 'C331 + C2O3 + 2*O2D2 + 3*HGA3 == Br'
            - 'C334 + N3P3 + 3*HGA3 + 3*HGP2 == Cs'
            - 'Pb == -2 * Br'
            - 'Pb == 2 * Cs'

    lennard_jones:
        - param: epsilon
          unit: kjmol
          frozen:
              guess: uff
        - param: sigma
          unit: nm
          Cs Cs: 0.459
          Cs Pb: 0.398
          Br Cs: 0.385
          Pb Pb: 0.623
          Br Pb: 0.314
          Br Br: 0.356
          C2O3 Cs: 0.458
          C2O3 Pb: 0.254
          Br C2O3: 0.252
          Cs O2D2: 0.202
```

```
            O2D2 Pb: 0.279
            Br O2D2: 0.349
            Cs N3P3: 0.383
            N3P3 Pb: 0.376
            Br N3P3: 0.344
            Cs HGP2: 0.391
            HGP2 Pb: 0.306
            Br HGP2: 0.244
            constraints:
                - 'Cs Cs   > 0.369'
                - 'Cs Pb   > 0.390'
                - 'Br Cs   > 0.358'
                - 'Pb Pb   > 0.603'
                - 'Br Pb   > 0.292'
                - 'Br Br   > 0.363'
                - 'C2O3 Cs > 0.438'
                - 'C2O3 Pb > 0.234'
                - 'Br C2O3 > 0.232'
                - 'Cs O2D2 > 0.182'
                - 'O2D2 Pb > 0.259'
                - 'Br O2D2 > 0.329'
                - 'Cs N3P3 > 0.363 '
                - 'N3P3 Pb > 0.356 '
                - 'Br N3P3 > 0.324 '
                - 'Cs HGP2 > 0.371 '
                - 'HGP2 Pb > 0.286 '
                - 'Br HGP2 > 0.224 '
            frozen:
                guess: uff

psf:
    rtf_file:
        - acetate.rtf
        - primary.rtf
    ligand_atoms: [C, O, H, N]

pes:
    rdf:
        func: FOX.MultiMolecule.init_rdf
        err_func: FOX.armc.err_normalized_weighted
        kwargs:
            -   mol_subset: !!python/object/apply:builtins.slice
                - -500
                - null
                - null
                atom_pairs:
                - [Cs, Cs]
                - [Cs, Pb]
                - [Br, Cs]
                - [Pb, Pb]
                - [Br, Pb]
                - [Br, Br]
                - [C2O3, Cs]
                - [C2O3, Pb]
                - [Br, C2O3]
                - [Cs, O2D2]
                - [O2D2, Pb]
                - [Br, O2D2]
```

```
    -   mol_subset: !!python/object/apply:builtins.slice
        - -500
        - null
        - null
        atom_pairs:
        - [Cs, Cs]
        - [Cs, Pb]
        - [Br, Cs]
        - [Pb, Pb]
        - [Br, Pb]
        - [Br, Br]
        - [Cs, N3P3]
        - [N3P3, Pb]
        - [Br, N3P3]
        - [Cs, HGP2]
        - [HGP2, Pb]
        - [Br, HGP2]

job:
  molecule:
    - acetateQMMD.xyz
    - primaryQMMD.xyz

  md_settings:
    template: qmflows.templates.md.specific.cp2k_mm
    settings:
      - prm: acetate.prm
        input:
          global:
            print_level: LOW
          force_eval:
            mm:
              poisson:
                periodic: xyz
                ewald:
                  ewald_type: spme
                  gmax: '62 62 62'
                  o_spline: 6
              forcefield:
                spline:
                  emax_spline: 50
                  #                         rcut_nb: 0.01
            subsys:
              cell:
                abc: '[angstrom] 100 100 100'
                periodic: xyz
          motion:
            print:
              cell:
                each:
                  md: 10
            md:
              ensemble: NVT
              temperature: 300.0
              timestep: 1
              steps: 10000
              thermostat:
                type: csvr
```

```
                csvr:
                  timecon: 1000
              print:
                energy:
                  each:
                    md: 10

      - prm: primary.prm
        input:
          global:
            print_level: LOW
          force_eval:
            mm:
              poisson:
                periodic: xyz
                ewald:
                  ewald_type: spme
                  gmax: '62 62 62'
                  o_spline: 4
              forcefield:
                spline:
                  emax_spline: 5
            subsys:
              cell:
                abc: '[angstrom] 100 100 100'
                periodic: xyz
          motion:
            print:
              cell:
                each:
                  md: 10
            md:
              ensemble: NVT
              temperature: 300.0
              timestep: 1
              steps: 10000
              thermostat:
                type: csvr
                csvr:
                  timecon: 1000
              print:
                energy:
                  each:
                    md: 10

monte_carlo:
  type: FOX.armc.ARMC
  iter_len: 50000
  sub_iter_len: 10
  logfile: armc.log
  hdf5_file: armc.hdf5
  path: ./
  folder: MM_MD_workdir
  keep_files: True
```


The best set of parameters obtained by means of this simultaneous fitting is shown in Table S10.

Table S10. Calculated Force Field Parameters for the simultaneous fitting of a $CsPbBr_3$ Perovskite NC Capped with Acetate and Methylammonium Ligands: Charge (e) and σ (nm)[a]

**Parameters**	
**Charges (NC core)**	
**Cs**	0.6316
**Pb**	1.2632
**Br**	-0.6316
**σ (NC core)**	
**Cs Cs**	0.543
**Cs Pb**	0.436
**Cs Br**	0.393
**Pb Br**	0.330
**Pb Pb**	0.603
**Br Br**	0.378
**Charges (Ligand Anchors)**	
**O2D2**	-0.4553
**C2O3**	0.4001
**N3P3**	-0.1642
**HGP2**	0.2167
**σ (Ligand Anchors to NC core)**	
**Cs C2O3**	0.483
**Pb C2O3**	0.250
**Br C2O3**	0.232
**Cs O2D2**	0.182
**Pb O2D2**	0.288
**Br O2D2**	0.360
**Cs N3P3**	0.441
**Pb N3P3**	0.383
**Br N3P3**	0.350
**Cs HGP2**	0.419
**Pb HGP2**	0.287
**Br HGP2**	0.258
